# Early Levallois and the beginning of the Middle Paleolithic in central Italy

**DOI:** 10.1371/journal.pone.0186082

**Published:** 2017-10-20

**Authors:** Sylvain Soriano, Paola Villa

**Affiliations:** 1 ArScAn, AnTET, CNRS, Université Paris Nanterre, Nanterre, France; 2 University of Colorado Museum, Boulder, Colorado, United States of America; 3 Istituto Italiano di Paleontologia Umana, Rome, Italy; 4 School of Geography, Archaeology and Environmental Studies, University of the Witwatersrand, Johannesburg, South Africa; Max Planck Institute for the Science of Human History, GERMANY

## Abstract

In the second half of the 19th century Pleistocene faunas were discovered in two sites, Sedia del Diavolo and Monte delle Gioie, contained in deposits of the Aniene River in the area of Rome (Latium, Italy). Fieldwork by A.C. Blanc in the late 1930’s proved the association of fauna and lithic industry within fluvial deposits interbedded with volcanoclastic layers. A human femoral diaphysis and a metatarsal were later identified in the faunal assemblage from Sedia del Diavolo and evaluated as Neandertal. The lithic assemblages from these two sites were the basis of the definition of the Protopontinian by M. Taschini, which she viewed as a late Middle Pleistocene industry very similar to the later, Upper Pleistocene Pontinian industries, thought to be characteristic of the Latium Mousterian. The chronostratigraphic framework of the Aniene river deposits has been recently updated and the lithic assemblages from these two sites are now confidently dated between 295 and 290 ka, close to the transition from MIS 9 to MIS 8. They fit chronologically between the industries of layers *m* and *d* from Torre in Pietra, a site 26 km northwest of Rome. The presence of the Levallois debitage is indisputable yet it occurs within an original technical context, different from what is known in other early occurrences of the Levallois. The date confirms the proposed chronology for the early Levallois in Europe. More importantly these two assemblages demonstrate that this technology can emerge in more diversified contexts than usually described. This suggests that its dispersal in Europe may have been rapid.

## Introduction

**Fig 1:**
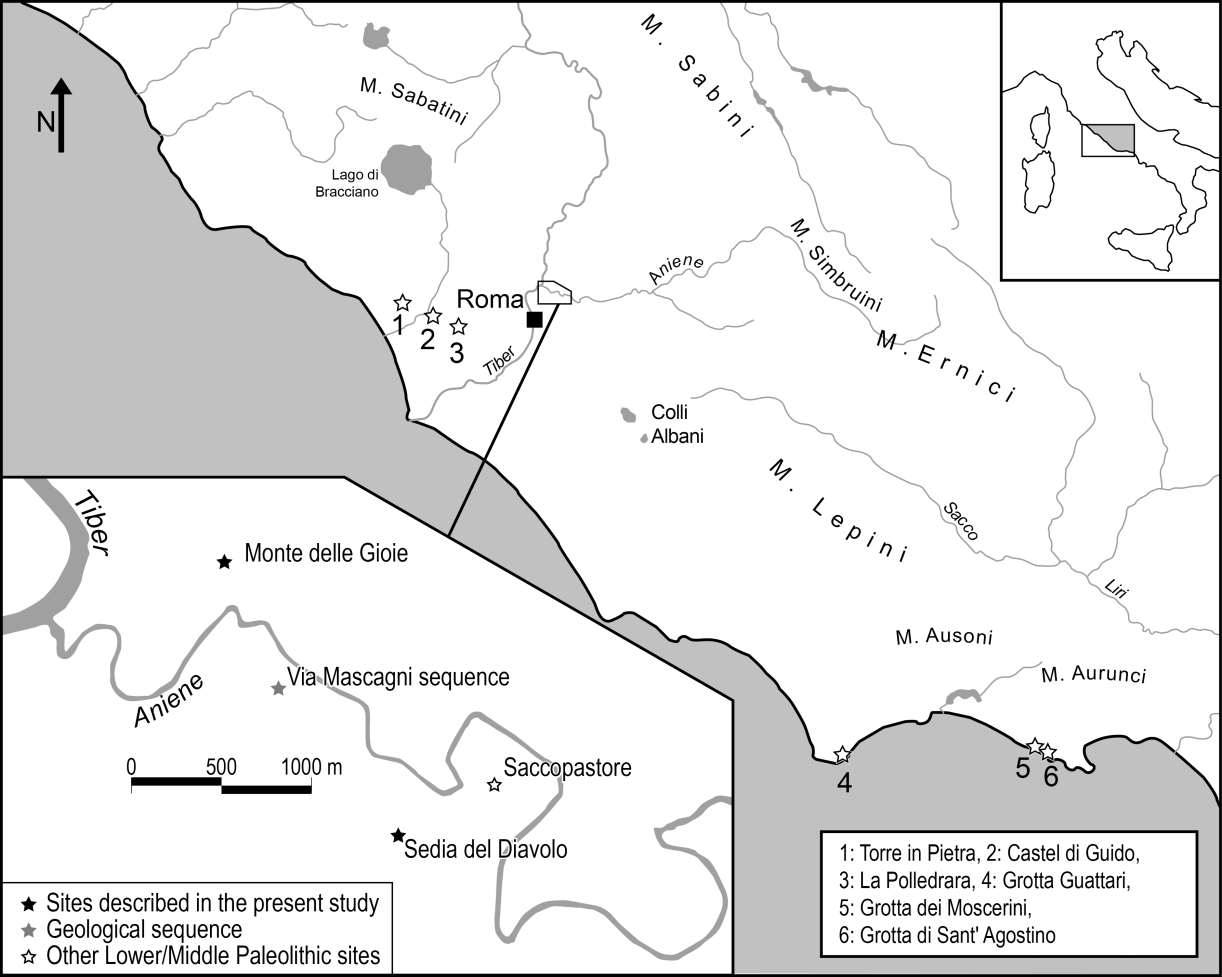
Location of Sedia del Diavolo and Monte delle Gioie (Central Italy) and other sites discussed in the text. Modified from Fig 3.1 in MOUSTERIAN LITHIC TECHNOLOGY: An Ecological Perspective by Steven L. Kuhn. Princeton University Press 1995.

Monte delle Gioie (hereafter MdG; 41°56'45"N; 12°30'44"E) and Sedia del Diavolo (SdD; 41° 55'46"N; 12°31'22"E) are the two oldest known archaeological sites within the city of Rome (Latium, Italy). They are located on the same middle terrace of the Aniene River, a left bank tributary of the Tiber, on opposite sides of the valley, in the north-east suburbs of Rome (Fig 1) They were discovered late in the 19th century and further surveyed and studied by A.C. Blanc from 1935 onward [1,2]. Blanc reported in detail the stratigraphic sequences, collected fauna and artifacts from both localities and established their contemporaneity (S2 File, History of Research). At both sites, the same layer of fluvial gravels yielded fossil faunas and lithic assemblages at the time considered Rissian in age [3–6]. According to Taschini [3] these assemblages, made on small flint pebbles, were characterized by a high proportion of standardized sidescrapers, mostly with Quina-like retouch, the presence of blanks with facetted platforms, and centripetal cores, a few with prepared platforms. The knapping techniques were direct percussion and the bipolar technique. According to Taschini these characters showed clear similarities to the Upper Pleistocene Mousterian industries in the Latium region also made on small flint pebbles, called Pontinian (e.g. Grotta dei Moscerini, Grotta Guattari and Grotta di Sant’Agostino; Fig 1). Thus she suggested that Sedia del Diavolo and Monte delle Gioie were the direct antecedents of the Pontinian and called them Protopontinian. The absence of Levallois debitage at both sites was noted by Piperno and Biddittu [7]. Their view was retained in the literature.

The stratigraphic section of Sedia del Diavolo based on work of Blanc and published by Taschini [3] showed the occurrence at the base of the sequence of a volcanic tuff, more than 25 m thick, called Tufo Lionato, now known to be an eruption event of the Alban Hills volcanoes, southeast of Rome (Fig 1). Above the fluvial gravels that contained fauna and lithic artifacts there was another pyroclastic flow with white pumices, now known to represent an eruptive event of the Monti Sabatini volcanoes, north of Rome. Thus two volcanic events bracket the archaeological level but their ages became known only more recently, when correlation of the local geologic terms with the Alpine sequence of glacial and interglacial periods was abandoned in favor of a more complex approach. The stratigraphic section of Monte delle Gioie is very similar to that of Sedia del Diavolo [5].

Since 1980 intensive research on the Middle and Upper Pleistocene structural setting and eruptive events of the Latium volcanic region developed an approach combining tectonic studies with fluvial and shallow water stratigraphy, the timing of sea-water oscillations and dating by the ^40^Ar/^39^Ar method the numerous volcanic events interbedded with other sediments. The dates show that fining-upward aggradational sequence of deposits occurred rapidly in response to sea-level changes due to glacial melting [8–14](S3 File).

**Fig 2:**
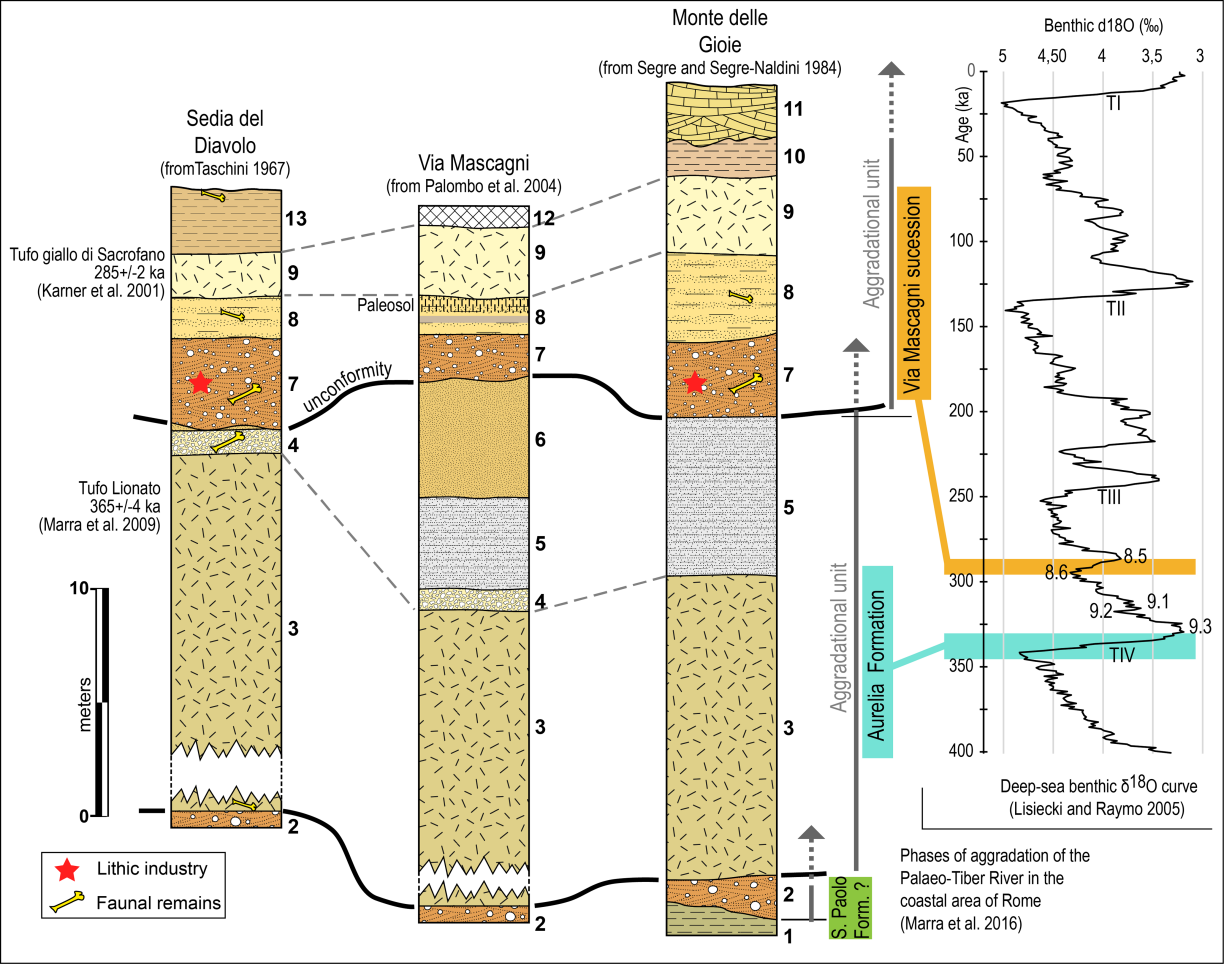
Stratigraphy of Sedia del Diavolo, Via Mascagni and Monte delle Gioie. 1: Clay with remains of conifer, 2: Fluvial gravels, 3: *Tufo Lionato*, 4: Fluvial epiclastic deposits, 5: Lacustrine silts, 6: Fluvial sands, 7: Fluvial gravels (with black pumice pebbles), 8: Lacustrine yellow silty sands with travertine and tuffite, 9: *Tufo giallo di Sacrofano*, 10: Marl with palustrean flora, 11: Travertine, 12: Modern backfill, 13: Colluvial deposits and post-glacial soil. Modified from [3,5,17]. The fluvial aggradational sequences are correlated to the deep-sea δ^18^O record from [20] (Data available at: http://lorraine-lisiecki.com/LR04stack.txt) according to [14]. TIV = Termination IV.

The geological setting of the lower Aniene River has been recently updated [14–16]. The outcrops studied by Blanc have been buried by the urban expansion of Rome but road construction close to the course of the Aniene River (Via Mascagni) opened new exposures and allowed further observation of Pleistocene deposits in the area of SdD and correlation of the SdD sequence with the one recognized in Via Mascagni [9,17]. The Tufo Lionato at the base of the SdD sequence is dated by ^40^Ar/^39^Ar to 365 ± 4 ka while the ash flow with pumices at the top, now known as Tufo Giallo di Sacrofano, is dated to 285 ± 2 ka [18,19]. Thus the aggradational sequence of Via Mascagni, SdD and MdG is clearly older than 285 ka and correlated by [15,16] with the glacial termination of MIS 8.6 and the following onset of warmer condition (MIS 8.5) (Fig 2; S3 File). As the deposition of the basal gravel is triggered by the maximum rate of sea-level rise during the glacial termination [16] fluvial gravels containing the industries were deposited during a narrow time span between the glacial maximum (at 295 ka according to [20]) and the glacial termination around 290 ka (According to [20] each glacial termination is dated by the temporal midpoint between the start and end of rapid change in δ^18^O). Thus the SdD and MdG lithic industry and human remains are assigned a tightly constrained age between 295 and 290 ka.

The aggradational sequence of Via Mascagni, Sedia del Diavolo and Monte delle Gioie is not represented in the Torre in Pietra stratigraphic sequence (26 km northwest of Rome) which contains in level *m* at its base an Acheulian assemblage now dated by ^40^Ar/^39^Ar and U-series to MIS 10 (c. 350 ka) and higher up an early Middle Paleolithic assemblage in level *d*, dated to between 270 and 240 ka, at the very beginning of MIS 7 [21]. Thus the industries from SdD and MdG are chronologically intermediate between the MIS 10 Acheulian and the early MIS 7 Middle Paleolithic of level *d* at Torre in Pietra. The artifacts and faunas from SdD/MdG, including two hominin remains from SdD assigned to Neandertals [22] (S1 File) are now confidently dated close to the transition from MIS 9 to MIS 8 between 295–290 ka and may be seen as a very old if not the oldest evidence of early Middle Paleolithic in Italy.

The objectives of this paper are (1) to describe the lithic industry recovered by A.C. Blanc at MdG and SdD; (2) to demonstrate that it is one of the earliest occurrence of Levallois in Italy; (3) to show that the Levallois method appears in a Middle Paleolithic type industry; (4) to point out that this industry is quite different from any early Levallois industry published so far and (5) to determine whether or not the industries from SdD/MdG could still be considered as Protopontinian as proposed by M. Taschini [3]. Our results will be discussed in the broader context of emergence and diffusion of the Levallois method in Western Europe.

## Material and methods

The SdD and MdG lithic assemblages are housed in the Pigorini National Museum of Prehistory and Ethnography in Rome and in the Italian Institute of Human Paleontology in the town of Anagni (Latium). Permits to study and take photos of the materials were obtained from the Soprintendenza of the Pigorini Museum (Prot. N. 212, 25.02.03/15, MBAC-S-MNPE Pigorini Cl. 25-02-04/5 and Cl. 23.03.02/1) and from Fabio Parenti, President of the Italian Institute of Human Paleontology.

Taschini inventoried 25 pieces from SdD (15 retouched pieces and 10 unretouched) and 62 retouched pieces and 30 unretouched from MdG. A single sidescraper from MdG illustrated by [3] was not found. In the Anagni sample from SdD, several pieces without evidence of human knapping or with dubious knapping features, with uncertain stratigraphic provenience or from later deposits were discarded. We recorded 25 pieces from SdD and 103 from MdG in our database.

As the two assemblages are small, we did not apply the sorting procedures used by us in the analysis of several assemblages from the Middle Stone Age [23–27] or the Lower and Middle Paleolithic [21]. Only fragments without knapping features and pieces too rolled to be studied were rejected. Retouched pieces and cores are assigned individual catalog numbers, unless already assigned by the Museum or the excavator, and are individually bagged in reusable zipper bags with pre-printed labels.

We developed a technological approach to reconstruct the reduction sequences from raw material procurement to tool manufacture [28,29]. Flakes produced through debitage were categorized with reference to flake productions usually encountered in the Western European Middle Paleolithic. Due to the small amount of pieces our study is mainly based on qualitative analyses including metrical, technological and typological attributes combined with particular attention to the sequence of manufacture.

## Results

### Assemblage composition

The total lithic assemblage from SdD and MdG is not large (N = 128; Table 1) but it is very diversified. Cores are frequent relative to the debitage (about 2.5 flakes per core). The frequency of retouched tools is high (56.2%) and most of them are sidescrapers. Retouched tools on flakes are dominant but 25% of them are made on cores and core fragments. Only four retouched tools are directly shaped on small pebbles or natural fragments.

**Table 1:** Counts of lithic pieces (retouched and unretouched) from Sedia del Diavolo and Monte delle Gioie.

		Core and core frag.	Flake and flake frag.	Pebble	Retouched natural fragment	Indet.	Total
Monte delle Gioie	Retouched	13	38	1	2	2	56
	Unretouched	15	32				47
Sedia del Diavolo	Retouched	5	9	1	0	1	16
	Unretouched	1	8				9
	Total	34	87	2	2	3	128

### Raw material and taphonomy

At MdG and SdD, flint is the dominant raw material with a single occurrence of chert or silicified limestone and 93.6% of the pieces with preserved cortical surfaces have water-worn cortex. In some later Mousterian sites from the area, active or fossil pebble beaches were the sources of flint pebbles [30]. However flint pebbles from marine beaches are usually more regular in shape than fluvial ones while the great majority of the pebbles knapped at both site have rather irregular or angular shapes. Thus the sources of raw material were very likely fluvial deposits.

The original assemblages have been depleted of the smallest elements by winnowing in the course of fluvial transport [31–33]. In fact, objects less than 20 mm are almost completely lacking but in Middle Paleolithic studies, the sorting threshold is usually set at 20 mm as smaller elements are not very useful for chaîne opératoire reconstruction. The assemblage also includes pieces with different states of preservation but the intensity or distance of transport may have been limited because the frequency of pieces with a high degree of fluvial abrasion is low [34]. In fact, 75.0% of the artifacts have fresh edges, without macroscopic evidence of fluvial transport and only 1.7% are very abraded (Table A in S1 File). The average size of lithic artefacts in both sites is rather similar (Table B in S1 File) indicating that the taphonomic biases and the possible recovery biases were identical on both sites. Since the goal of our study is primarily an understanding of technology we believe that the impact of taphonomic biases on our study are minor. Thus the assemblages from SdD and MdG, originating from the same fluvial deposits of the Aniene River and showing the same fluvial transport features (size sorting, degree of abrasion) were merged for the present study. Great similarities between these assemblages were emphasized by previous scholars [3,35,36].

### An early Middle Paleolithic technology

**Fig 3:**
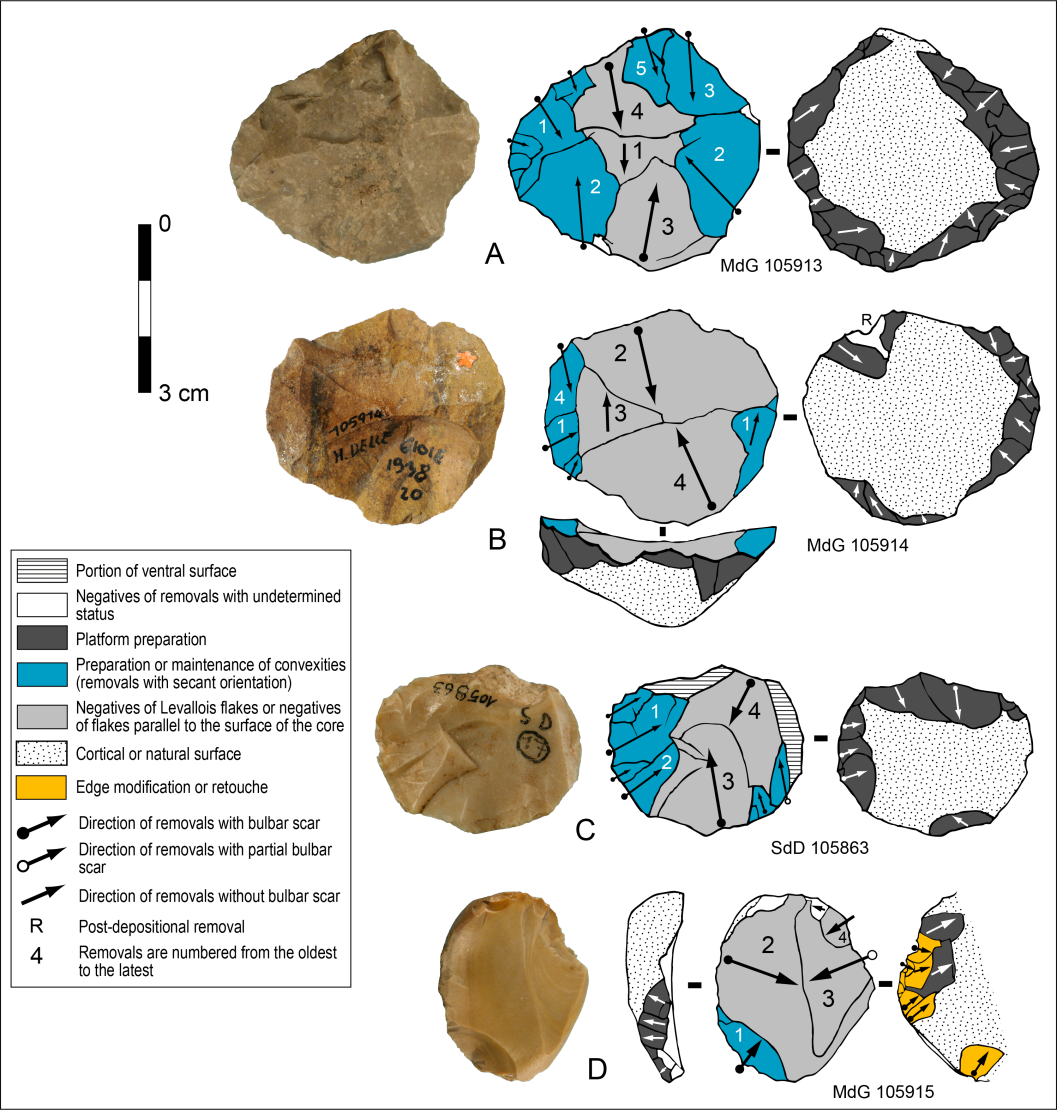
Sedia del Diavolo / Monte delle Gioie. Cores. (A-C) Levallois cores. (D) Possible Levallois core. (B: photo by F. Naccari of the Pigorini Museum).

At least three types of chaîne opératoire have been used to produce blanks. A fully-fledged and formalized Levallois technology is the most characteristic component of the SdD and MdG assemblages but it is not dominant. Three cores with a recurrent centripetal exploitation are undoubtedly categorized as Levallois (Fig 3A–3C). They are all fitting the criteria proposed by Boëda [37–39], Inizan et al. [29], Brantingham and Kuhn [40]:

1. the core is shaped by means of two intersecting flaking surfaces;
2. the two surfaces are convex, asymmetrical and hierarchically related, one is the debitage surface, from which products are removed, the other constitutes the striking platform. The two surfaces are not interchangeable;
3. the debitage surface is shaped so that the morphology of the product is pre-determined, which is essentially a function of the lateral and distal convexities of the surface;
4. the Levallois products (filled in light grey on Fig 3) are split off along a fracture plane that is parallel or sub-parallel to the plane of intersection of the two surfaces, they are spanning the middle or most of the debitage surface;
5. the convexities are shaped and maintained with short removals, centripetal and oblique with respect to the plane of intersection (filled in blue on Fig 3) and;
6. the striking platform size and shape is adjusted to allow removal of flakes parallel to this plane, usually through retouch or faceting.

The chronology of removals on cores A and B clearly shows that the extraction of Levallois flakes is preceded by preparation of convexities and also at times followed by a partial re-preparation. This alternation is typical of the Levallois volumetric conception. Core C was prepared on a flake, a small portion of its of ventral surface, is still visible due to the shortness of the reduction sequence.

Two cores with hierarchically organized surfaces and centripetal removals might be Levallois but they are lacking or have a limited number of small removals to control peripheral convexities in their final condition (Fig 3D). A core fragment also has centripetal scars.

**Fig 4:**
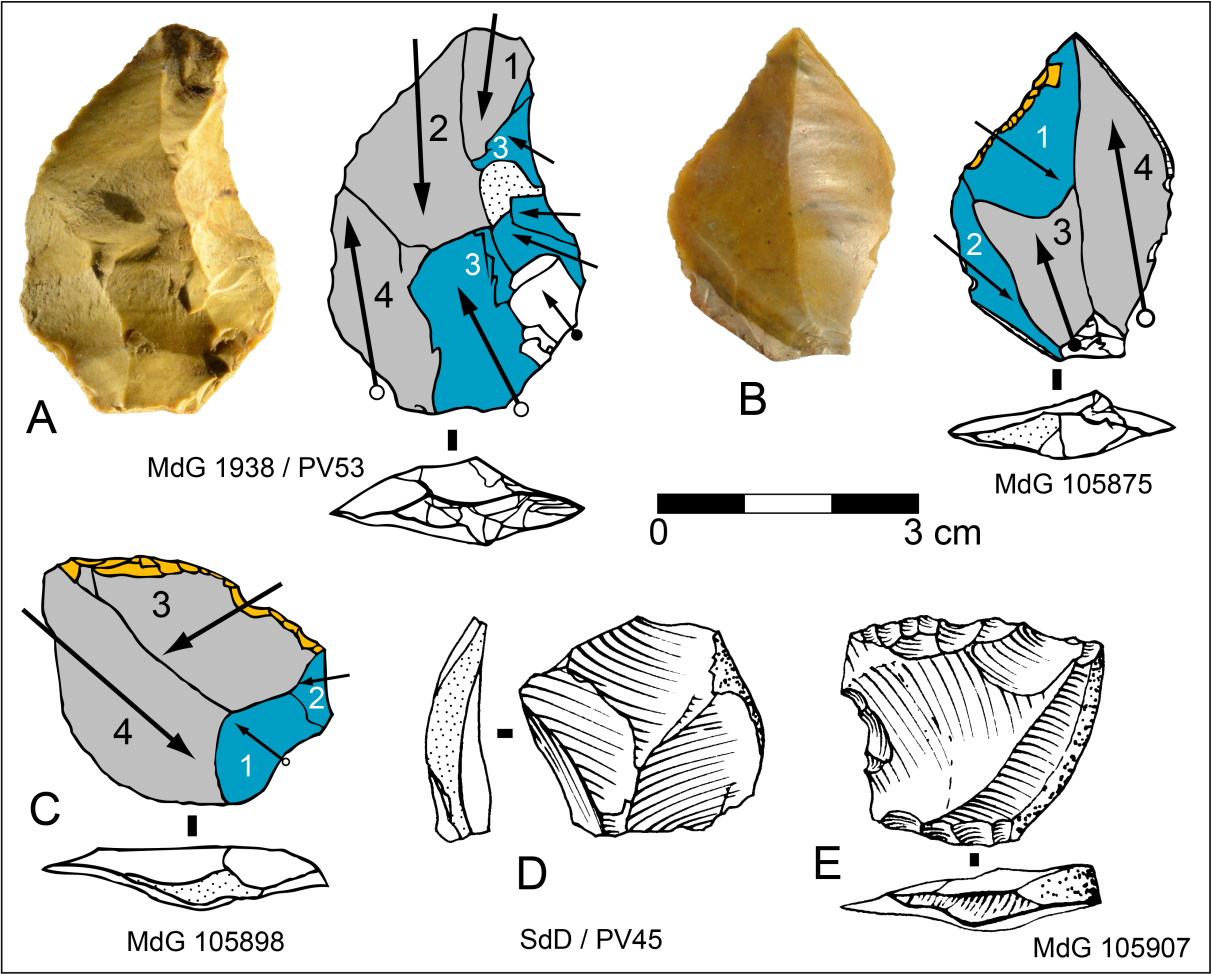
Sedia del Diavolo / Monte delle Gioie. Levallois and centripetal flakes. (A-C) Levallois flakes. (D-E) Centripetal flakes that might have been removed from a Levallois core. Caption and symbols used in drawings as in Fig 3. (B: photo by F. Naccari, Pigorini Museum).

On a few flakes (N = 5) the organization of scars fits with our description of Levallois cores, thus they have been categorized as Levallois flakes (Fig 4A–4C). Short, oblique scars with respect to the ventral surface along a lateral edge are remains of convexities management. Platform preparation was typically faceted (Fig 4A) or surprisingly unprepared -a natural surface- (Fig 4B) probably because its orientation was suitable for flaking. Only marginally retouched edges have been observed on these few Levallois flakes.

**Fig 5:**
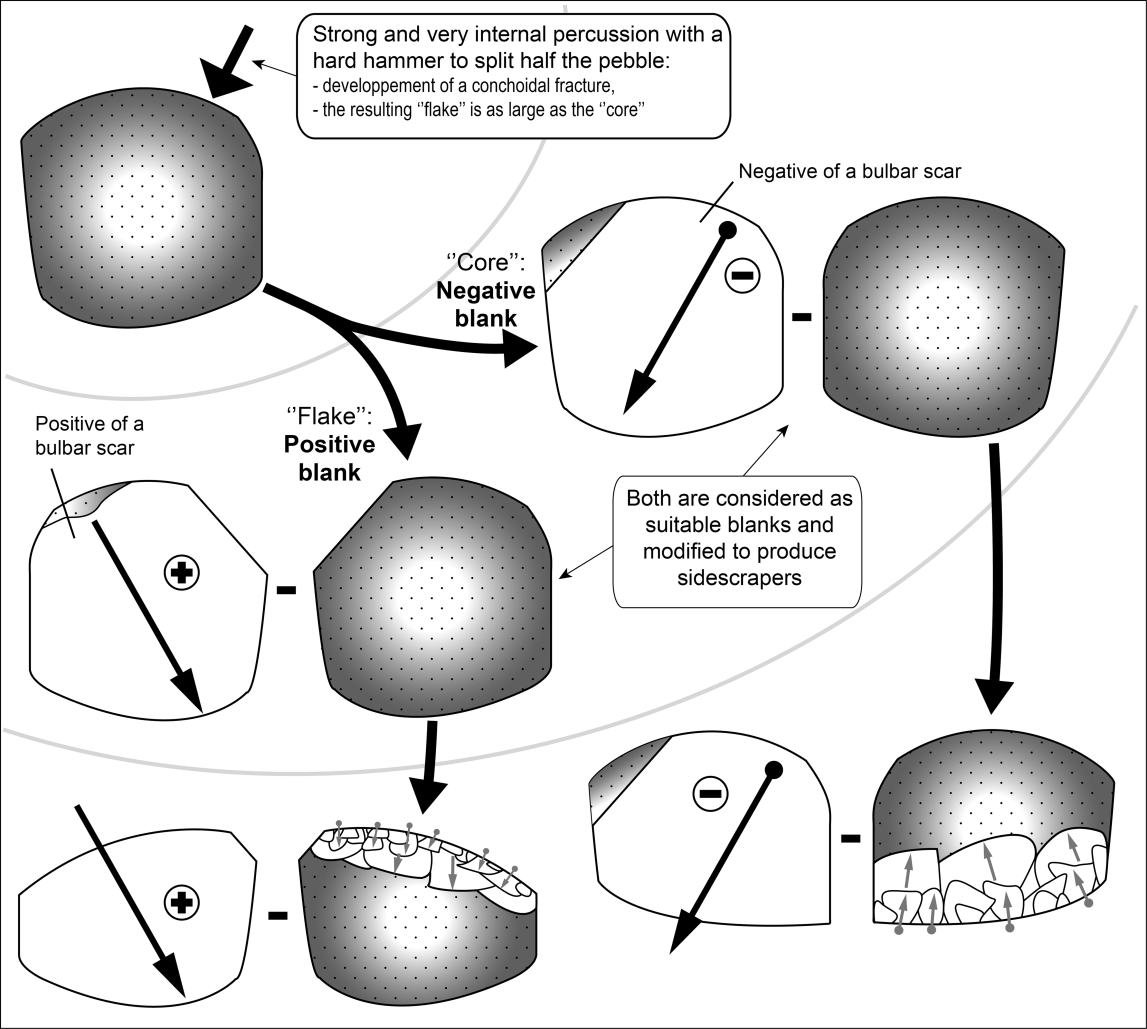
Schematic representation of the original chaîne opératoire used by the Sedia del Diavolo and and Monte delle Gioie’s flintknappers to produce large blanks retouched as sidescrapers.

A second, very distinctive and original, chaîne opératoire, between debitage and shaping, was used at SdD and MdG. Small rounded flint blocks or cobbles were split in half by means of a strong and very internal percussion with a hard hammer (Fig 5). Nevertheless the mode of fracture remains conchoidal in most of the cases and one can distinguish a ‘core-like’ piece (negative blank) with a single invasive negative scar and an entirely cortical thick ‘flake’ (positive blank). Bipolar percussion on an anvil was not used because the fracture is clearly conchoidal without any crushing and splintering on the distal end [25,41–44]. The aim is to obtain two blanks, almost equal in size, as large as the initial raw material volume and with a flat surface almost as large as the pebble itself. Structurally the two blanks are similar.

**Fig 6:**
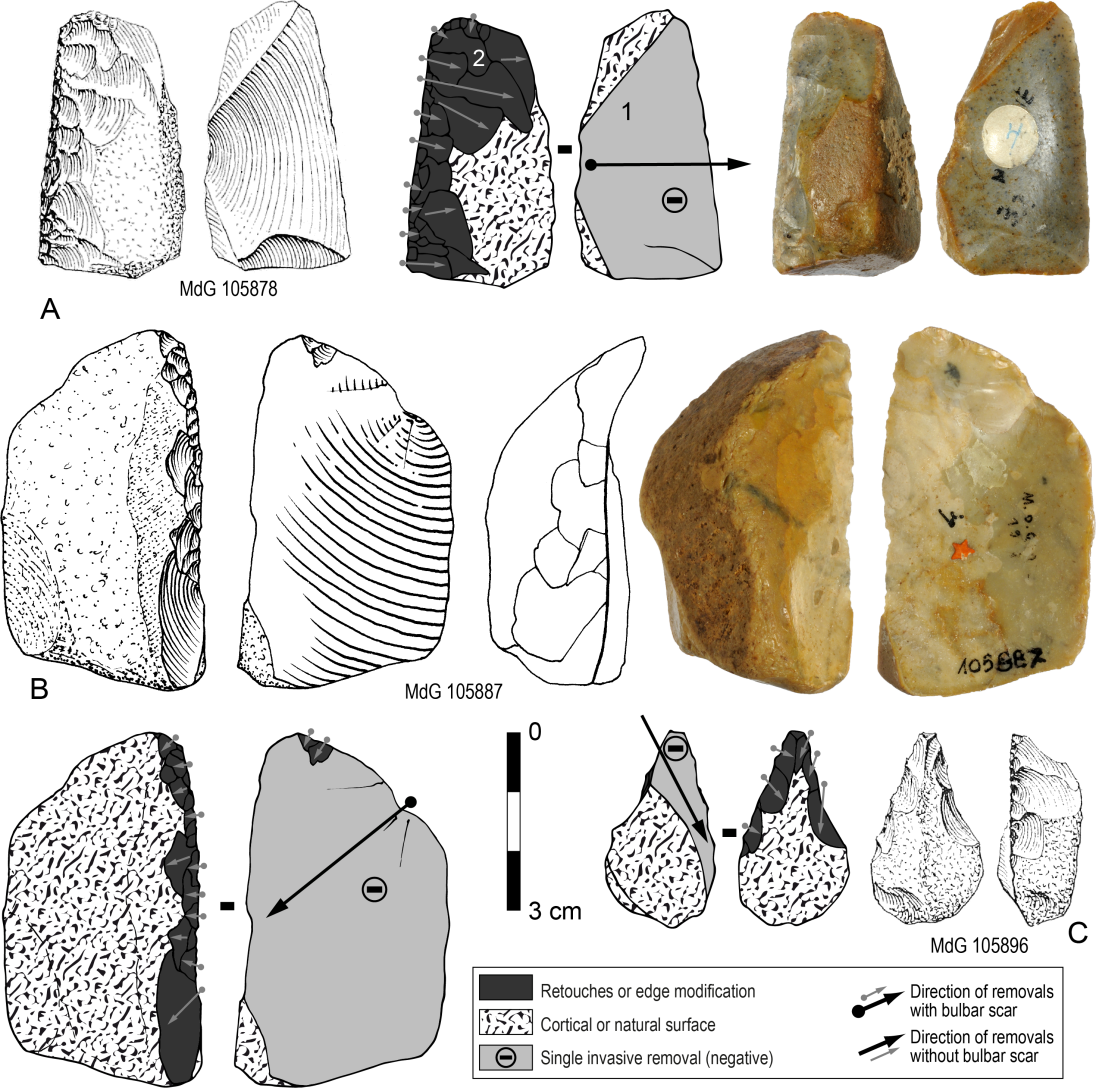
Sedia del Diavolo and Monte delle Gioie. **Retouched core-like pieces.** (A-C) « Core-like » pieces with a single invasive removal used as blanks for sidescrapers (A, B) or convergent scraper (C). (A, B: photos by F. Naccari, Pigorini Museum). Line drawings A and C modified after [3].

**Fig 7:**
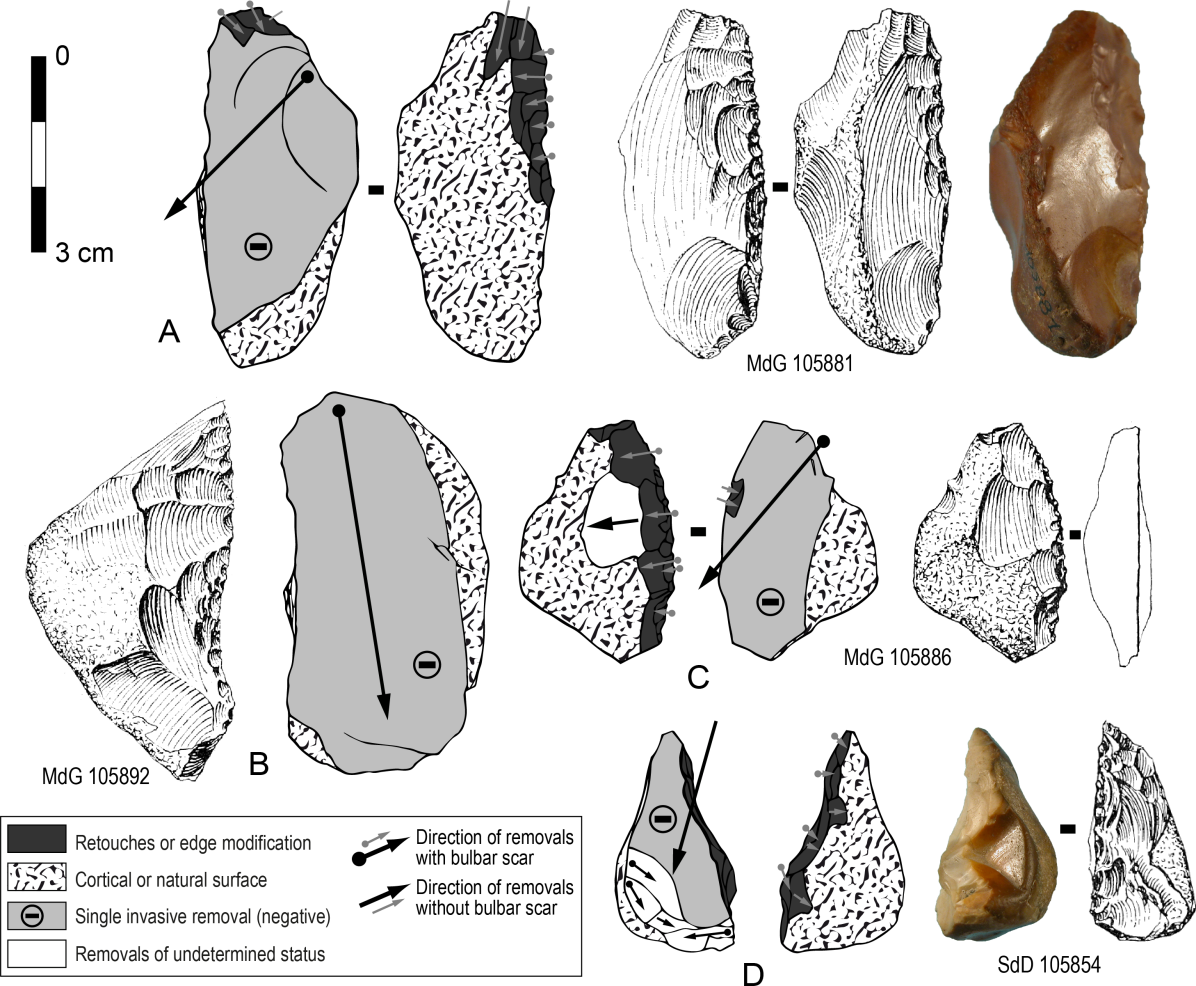
Sedia del Diavolo and Monte delle Gioie. **Retouched core-like pieces.** (A-D) « Core-like » pieces with a single invasive removal used as blanks for sidescrapers. Line drawings modified after [3].

**Fig 8:**
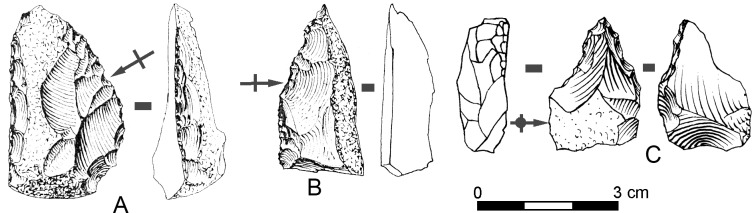
Sedia del Diavolo and Monte delle Gioie. **Scrapers on cortical flakes.** (A-C) Cortical flakes [transformed in sidescrapers (A, B) or *déjeté* scraper (C)] that are possible complements of « Core-like » pieces with a single invasive removal. The platform was removed on A and B. Line drawings A and B modified after [3].

At MdG and SdD both flake and core resulting from this chaîne opératoire were considered suitable blanks. Once produced they were systematically retouched as sidescrapers (or convergent scrapers) and no blanks from this production seem to have been left unretouched. ‘Cores’ of that type were easy to identify (N = 11; 15.2% of retouched tools) because the retouched edge was commonly opposed (Fig 6A and 6B; Fig 7A and 7C) or adjacent (Fig 7B) to the negative of the bulbar scar. In some cases, especially highly reduced tools, the negatives of bulbar scar have been removed by one or two retouched edges (Fig 6C; Fig 7D). These scrapers are variable in size, especially thickness, from very thick (Fig 7B) to thinner ones (Fig 7C) in relation to their length and width. Flakes produced through this chaîne opératoire are much more difficult to identify with confidence because they are just totally cortical flakes and because their proximal part has been frequently removed due to reduction (Fig 8). They might also be thinner than cores.

**Fig 9:**
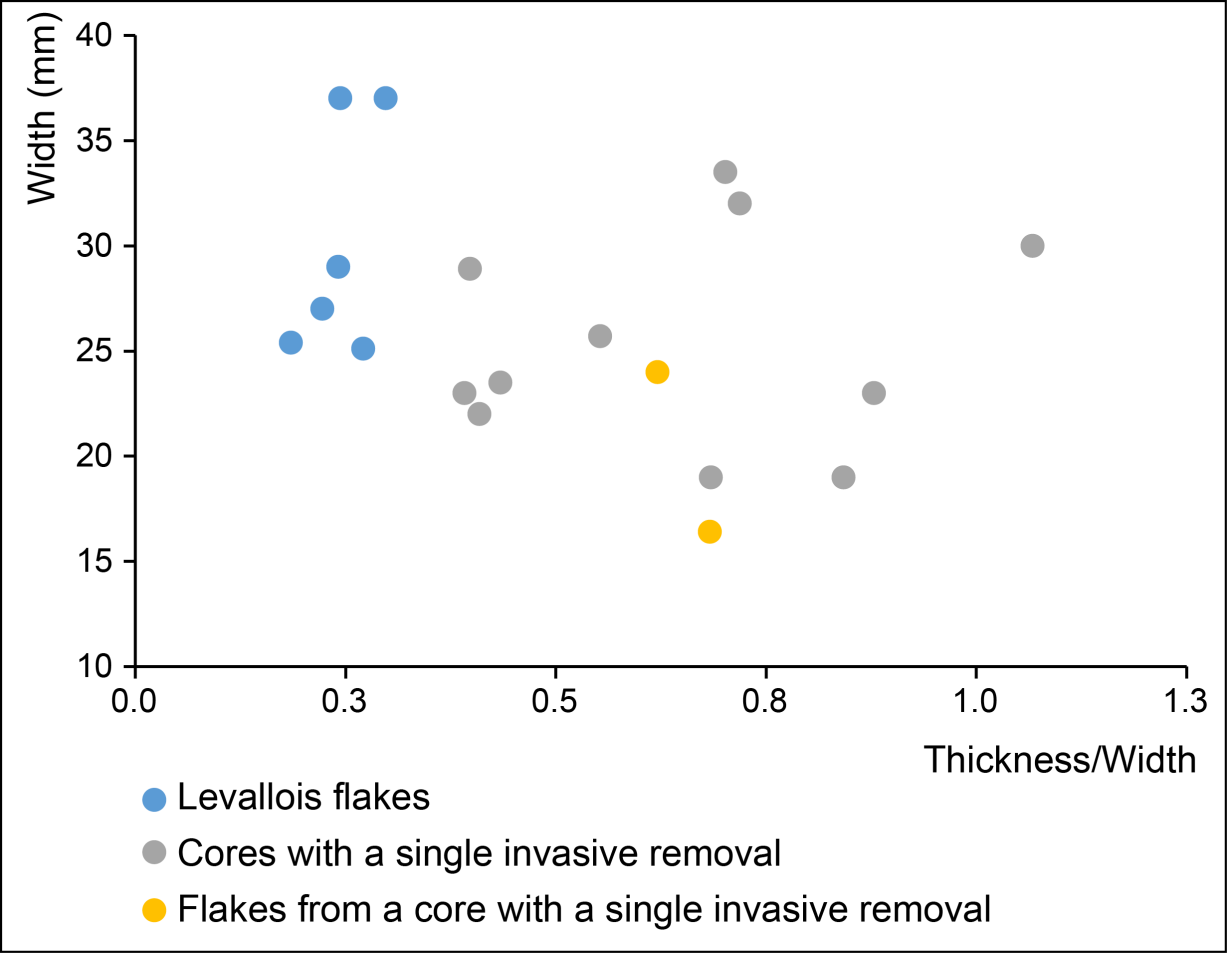
Scatter diagram of width against thickness/width of Levallois flakes, retouched ‘core-like’ pieces and retouched flakes from ‘core-like’ pieces. Levallois flakes are thin blanks whose size variations are proportional (their thickness/width ratio varies little). Negative or positive blanks from the ‘core-like’ production system are thicker (nearly as thick as wide for heavily retouched ones) but much more variable in size.

Blanks produced through this method are totally different from Levallois blanks and probably functionally complementary. They are significantly thicker than Levallois blanks (Fig 9) and consequently several of the sidescrapers made from these blanks bear a scaled retouch close to the classical sidescrapers of the Quina Mousterian. Superposition of many cycles of retouch is responsible for the stepped morphology of some scrapers’ edges (Fig 6C and Fig 7D).

This chaîne opératoire was never described before. We failed to find in the literature an exactly similar way to produce and modify two thick cortical blanks per nodule. At Les Tares, an Early Middle Paleolithic site in South-Western France, an experimental study has shown that a very internal percussion performed with hard hammer when the contact of the hammer with the core is not through a rounded end but rather through a longer edge of the hammer, produces very thick flakes, as large as the raw material volume, but the fracture was almost non-conchoidal [45] and usually several flakes were produced per nodule.

In assemblages of the Lower and Middle Paleolithic of the Latium a few small tools were made by using direct hard hammer percussion to flake a pebble with a large, unique and invasive removal. The large surface of the removal on the "core" or the "flake" was used as a striking platform for retouch. In the Italian Acheulian from Castel di Guido a few tools were made on core-like blanks (negative blanks in [21]: fig 20) but they are irregularly modified with steep retouch. They are lacking in the Acheulian from Torre in Pietra level *m*. In Middle Paleolithic industries from Torre in Pietra level *d* ([21]: fig 24) and Grotta dei Moscerini these core-like blanks are also present but do not occur frequently (respectively 3.3% and 2.5% of retouched tools). They are more finely retouched (sidescrapers) than at Castel di Guido. At Torre in Pietra level *d* invasive removals were produced by direct freehand percussion (conchoidal fracture) whereas at Moscerini both conchoidal and non-conchoidal fracture (on anvil) occur. Core-like pieces were more commonly produced and used as blanks for retouched tools by the flintknappers of SdD and MdG than in the sites listed above.

**Fig 10:**
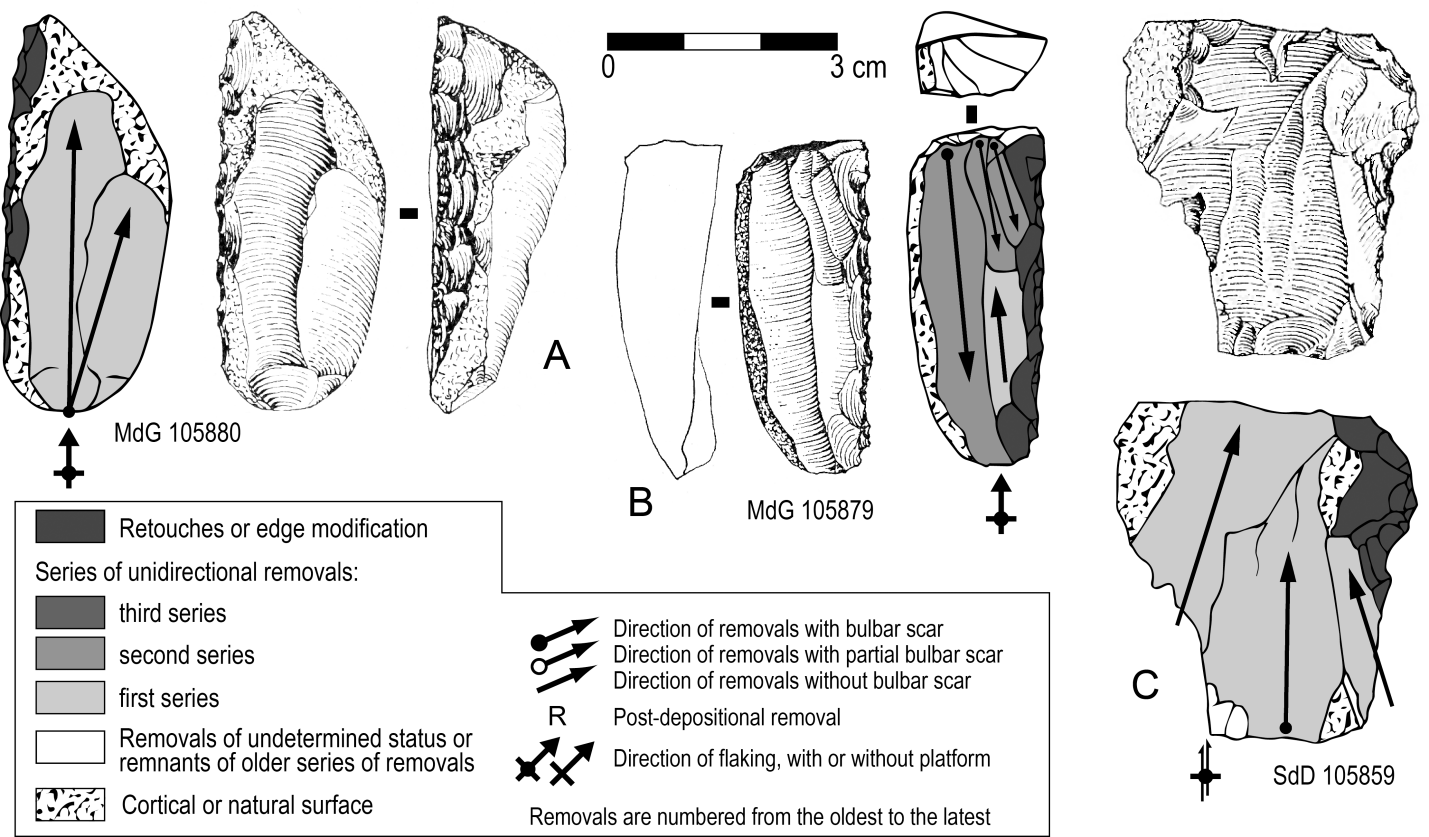
Sedia del Diavolo and Monte delle Gioie. **Retouched end-products with scars of successive series of unidirectional parallel removals.** Line drawings modified after [3].

**Fig 11:**
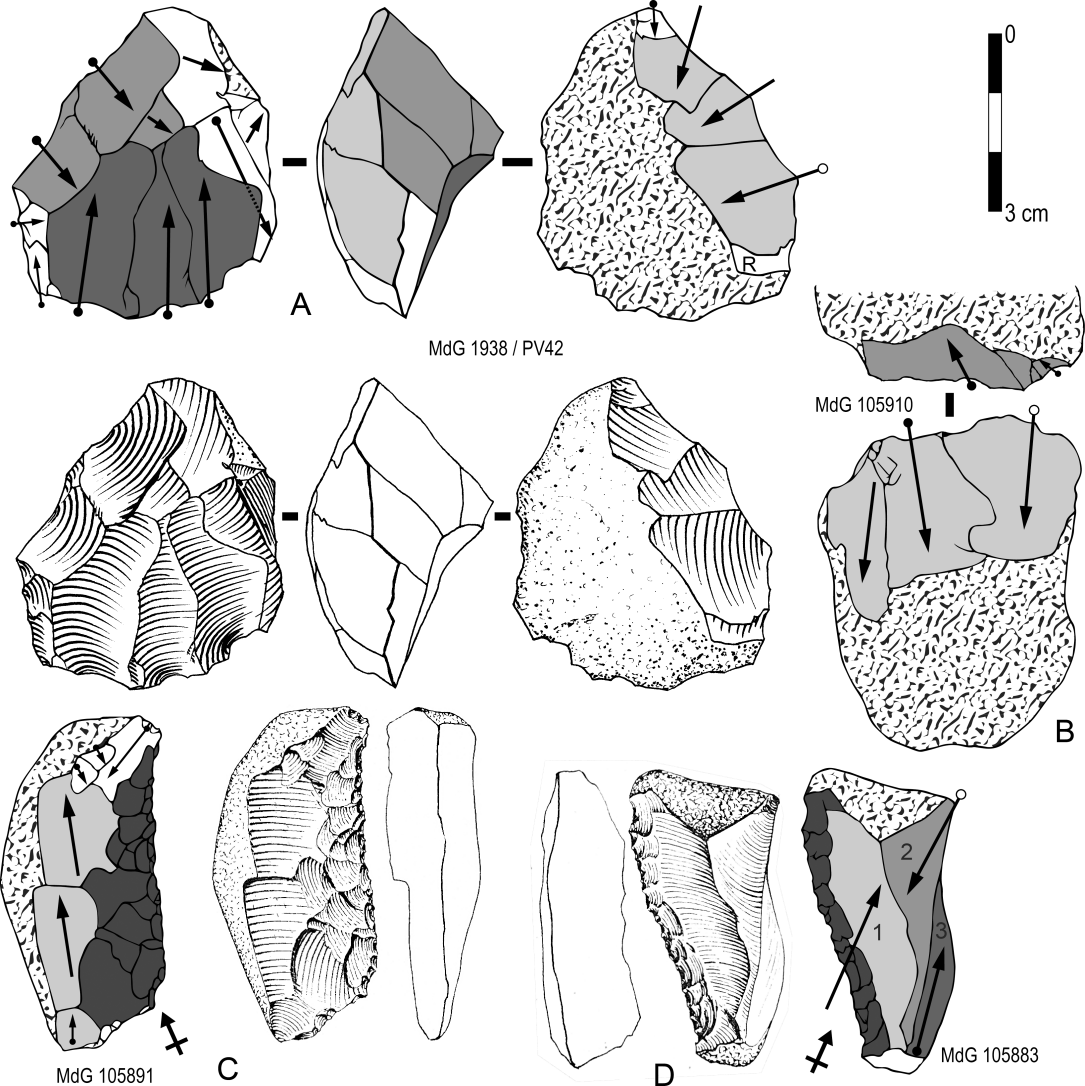
Sedia del Diavolo and Monte delle Gioie. **Cores (A, B) and retouched end-products (C, D) with scars of successive series of unidirectional parallel removals.** Caption and symbols used in drawings as in Fig 10. Line drawings C and D modified after [3].

A third reduction sequence documented by end-products with scars of successive series of unidirectional parallel removals (Figs 10 and 11) indicates a more conventional scheme to produce blanks (N = 14). Series of removals were flaked from unprepared cores (Fig 11A and 11B) hence remains of cortex or natural surfaces appear on most blanks (Fig 10, Fig 11C and 11D). This scheme can induce high variations in core morphology depending on how the series of removals are organized ([32]: fig 10). Here, in most of the cases, a single platform or two opposed platforms were used according to the organization of scars on the blanks. Among the few cores (N = 4) a single one has three series of unidirectional removal scars and older negatives with unclear pattern are also visible (Fig 11A) whereas the others have a simpler pattern or were exploited with a shorter chaîne opératoire (Fig 11B). Twelve of the fourteen blanks have been retouched mostly as sidescrapers.

### Secondary flaking

The repeated use of secondary flaking (called ‘ramification’ in French [46,47]) is one of the features that defines the SdD and MdG industry as Middle Paleolithic. Secondary flaking is not an exclusive feature of the Middle Paleolithic as it was already described in earlier industries [46,48]. Nevertheless, secondary flaking is a form of blank production that occurs more frequently in the Middle Paleolithic echoing the diversification of flaking methods [49]. In the industry from High-Lodge, now considered to be early Middle Paleolithic in age [50], N. Ashton [51] concludes that in the majority of cases the flaked flakes should be regarded as tools whereas secondary flakes were very rarely retouched. Secondary flakes of so-called Le Pucheuil-type [52] from the early Middle Paleolithic site of Le Pucheuil in Normandy (France) were also unretouched but use-wear analysis shows that they were used for multiple purposes [53]. Secondary flaking to produce supplementary blanks for retouched or unretouched tools has been documented in numerous though not all Middle Paleolithic industries of Western Europe. In Quina Mousterian industries scrapers with scaled retouch (Quina-type retouch) were frequently re-flaked to produce small blanks themselves retouched as side or transverse scrapers [46,54,55].

**Fig 12:**
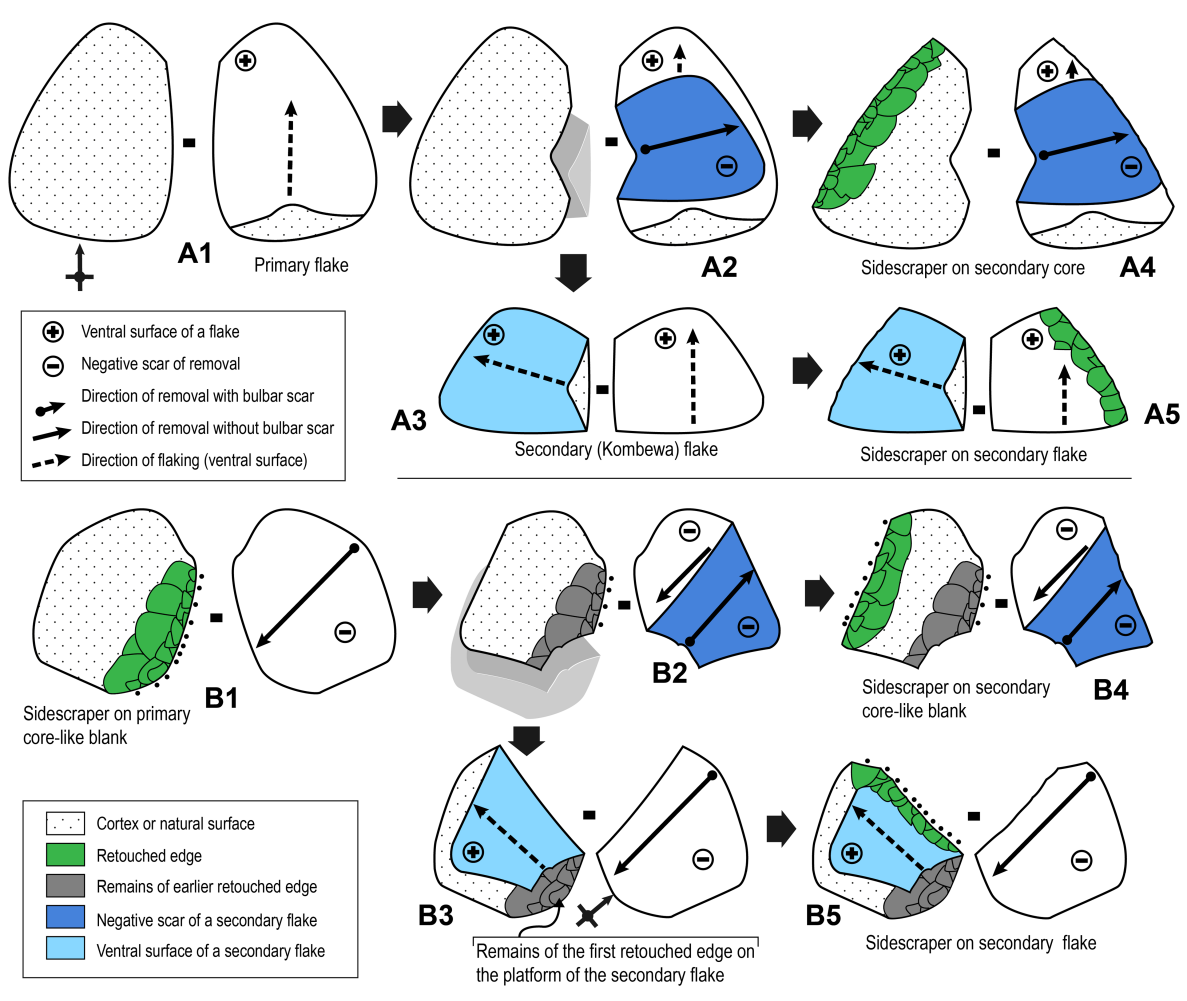
Schematic description of secondary flaking observed in the industry from Sedia del Diavolo and Monte delle Gioie. (A1-A5) First modality. Secondary flaking on a flake (Kombewa modality). (B1-B5) second modality. Secondary flaking on a core-like scraper. In both modalities, cores and flakes were retouched as sidescrapers.

**Fig 13:**
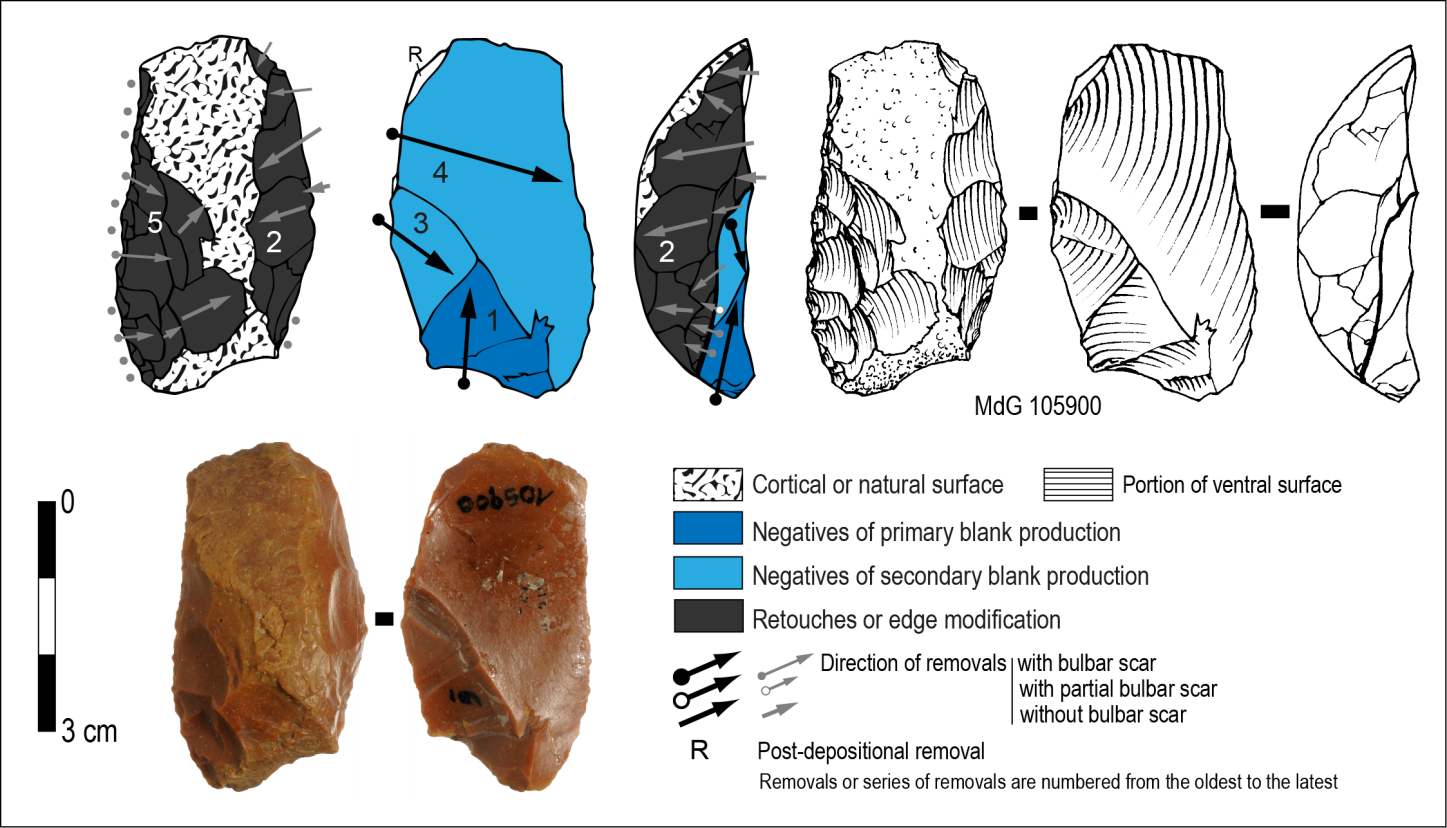
Monte delle Gioie. Retouched (sidescraper) secondary core made from a sidescraper shaped on a primary core-like piece.

Six artifacts from SdD/MdG show evidence of secondary flaking according to two different modalities (Fig 12). In each case the blanks produced in this manner were retouched. The first modality, called Kombewa (Modality 1 in [56]), consist in extracting a flake, more or less invasive, from the ventral face of a primary flake (Fig 12A1–12A5). This secondary flake (2 artifacts in our case) as well as the secondary core (i.e. the flake used as core; 1 artifact) were retouched as sidescrapers or denticulates. The second modality involves the core-like pieces produced by the second reduction sequence described above (Fig 12B1–12B5; Fig 13). A percussion applied to one of the retouched edges of a scraper extracts a flake from the flat surface of the core-like piece. Note that the secondary flake and even the already retouched core-like piece were retouched afterward. In a single case a secondary flake was extracted from the dorsal face of a first flake (Nahr-Ibrahim modality, Modality 2 in [56]). As in the previous case, the primary flake was retouched prior to the extraction of the secondary flake and was retouched again afterward. As could be expected four cases of secondary flaking concern a reddish-brown flint which has the finest texture in the assemblage.

**Fig 14:**
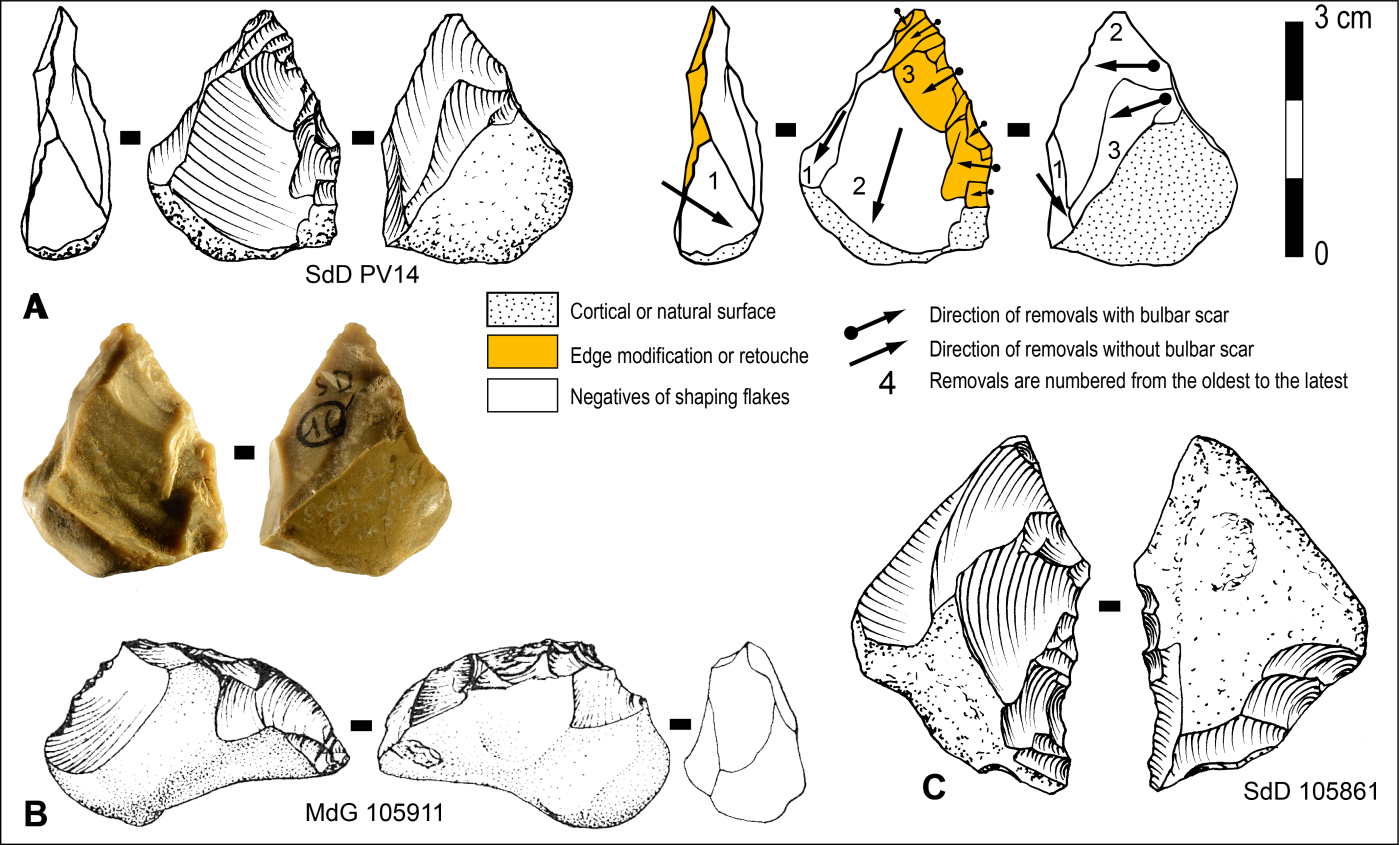
Sedia del Diavolo and Monte delle Gioie. **Bifacial tools shaped on small flint pebbles. (**A) Bifacial sidecraper. A flat surface is first shaped then the opposite one is retouched. (B-C) Bifacially retouched tools. Line drawing of B modified after [3].

The shaping of small pebbles to directly make retouched tools is the least represented reduction sequence at SdD/MdG. Three artifacts were produced in this manner (Fig 14) but only one has a regularly retouched edge (Fig 14A).

The distribution of tools types at MdG and SdD (Table 2) is in agreement with Middle Paleolithic patterns. Simple scrapers dominate the tool-kit followed by pieces with irregular retouch (abrupt retouch, short or marginally retouched edges), tools with denticulated edges and tools with convergent retouched edges (convergent and *déjeté* scrapers, Tayac point). It was considered that the high frequency of retouched tools (56.2%, i.e. 72/128 in Table 1), especially scrapers (Table 2), was a recovery bias [36]. It is now explained by the original chaîne opératoire described here which produces two cortical blanks for scrapers without any byproducts.

**Table 2:** Sedia del Diavolo and Monte delle Gioie. Blanks of retouched tools.

	Flakes	Cores	Shaped pieces	Natural	Indeter- minate	Total	%
A- Simple scrapers	26	12	1	2	1	42	*58*.*3*
B- Double scrapers	2	1	0	0	0	3	*4*.*2*
C- Convergent retouched edges	3	2	0	0	0	5	*6*.*9*
D- Denticulated edges	3	1	1	0	0	5	*6*.*9*
E- Notches	2	1	0	0	0	3	*4*.*2*
F- Miscellaneous	1	0	0	0	1	2	*2*.*8*
G- With irregular retouch	10	2	0	0	0	12	*16*.*7*
Total	47	19	2	2	2	72	

Tool types are classed as follows: A- Simple, transverse, inverse and bifacial sidescrapers; B- Double and alternate sidescrapers; C- Convergent and déjeté sidescrapers, Tayac points; D- Denticulate, denticulated endscraper and bifacial denticulate; E- Retouched notches, Clactonian notches; F- Miscellaneous: Beak, scaled pieces: G- Pieces with abrupt retouch, short or marginally retouched edges. Composite tools are included in the quantitatively dominant type.

## Discussion

Fifty years after the publication of Sedia del Diavolo and Monte delle Gioie by Mariella Taschini [3] our research has allowed us to describe the technological structure of the two assemblages and to show their originality. Clearly it is the Levallois technology that distinguishes these assemblages from earlier ones, whether the Acheulian of Torre in Pietra level *m* [21] or assemblages without bifaces, such as La Polledrara [57–59].

### The appearance of the Levallois technology

We agree with White et al. [60] that focusing discussions on Levallois technology may bring us to underestimate the role and importance of other prepared core technologies, but confident identification of Levallois is still a key point for the study of the emergence of Middle Paleolithic traditions. As pointed by Malinsky-Buller [61], in Lower and Middle Paleolithic industries some cores can be confused with Levallois cores because they share some but not all characters. Such cases have been rightly described as ‘simple prepared-core technology’ [62] and these cores can be encountered in assemblages predating the appearance of Levallois, as for instance at Cagny-la-Garenne [63] or in association with the earliest Levallois cores. This may explain persistent discrepancies in publications about the timing of appearance of the Levallois technology.

The chronology of the appearance of Levallois production in Western Europe have been recently reviewed in several papers [64–68]. Pre-MIS 9 occurrences of the Levallois method listed in these papers are usually considered dubious for two reasons: (1) Levallois-like items are very rare and might result from technical convergence and/or (2) their chronology is insecure. SdD/MdG fits with a dozen of convincing West-European sites with Early Levallois spanning from (late?) MIS 9 to early MIS 8.

The archaeological record from Spain is a case in point. Occurrence of the Levallois method has been reported from the middle stratigraphic unit of Ambrona comprising layers excavated by F.C. Howell and L. Freeman between 1960 and 1983 (called Upper Complex in the central and eastern sector of the site) and layers excavated by M. Santonja and A. Pérez-González between 1993 and 2000 in the Middle Stratigraphic Member, unit AS6 in the eastern sector [69,70]. Levallois cores are recorded for the Howell and Freeman's series and Levallois flakes but no cores are reported for the smaller series of the Santonja and Pérez-González's excavation. The industry of the Middle Stratigraphic member is in marked contrast with the Lower Complex where there is no evidence of the use of the Levallois method [70,71]. ESR and U-series combined on horse teeth from AS6 have produced two date: 366 +55/-51 and 314 +48/-45 ka [72]. For the lower levels (AS1 and AS2 of the Lower Stratigraphic Complex) dates are much too young. The problems created by environmental conditions resulting in unacceptable dates for the Acheulian of the Lower Complex are discussed in [73,74] who suggests a date, based on a mean value, of 350 ka for AS6, equivalent to MIS 10. This chronology is also supported by morphostratigraphic position of the site, and with other dates obtained in nearby localities [75]. Such an early age would seem to require further discussion as it predates most if not all confident occurrence of Levallois debitage in Western Europe.

In Italy, secure evidence of Levallois production is recorded at San Bernardino Cave at the end of MIS 7 [65] and earlier at Torre in Pietra layer *d* now dated to the early part of MIS 7 between 270 and 240 ka [21]. At Cave dall’Olio, the MIS 9 age of Levallois finds buried in a thick fluvial terrace is argued through correlation with the long paleosoil-sedimentary record from the Po Plain [76] but this chronological framework is not supported by any geochronological dating and must be considered with caution. The site from Guado San Nicola is claimed to have the earliest occurrence of Levallois technology in Italy within an Acheulian industry of late MIS 11 age [77] thus pushing back of almost an entire glacial cycle the appearance of Levallois technology in Italy [65,76] and more widely in Western Europe [68]. According to [77] the transition from the Lower to the Middle Paleolithic would have occurred significantly earlier than generally considered and this is challenging our results on SdD and MdG. Clearly the identification of Levallois technology is a key point in the debate.

**Fig 15:**
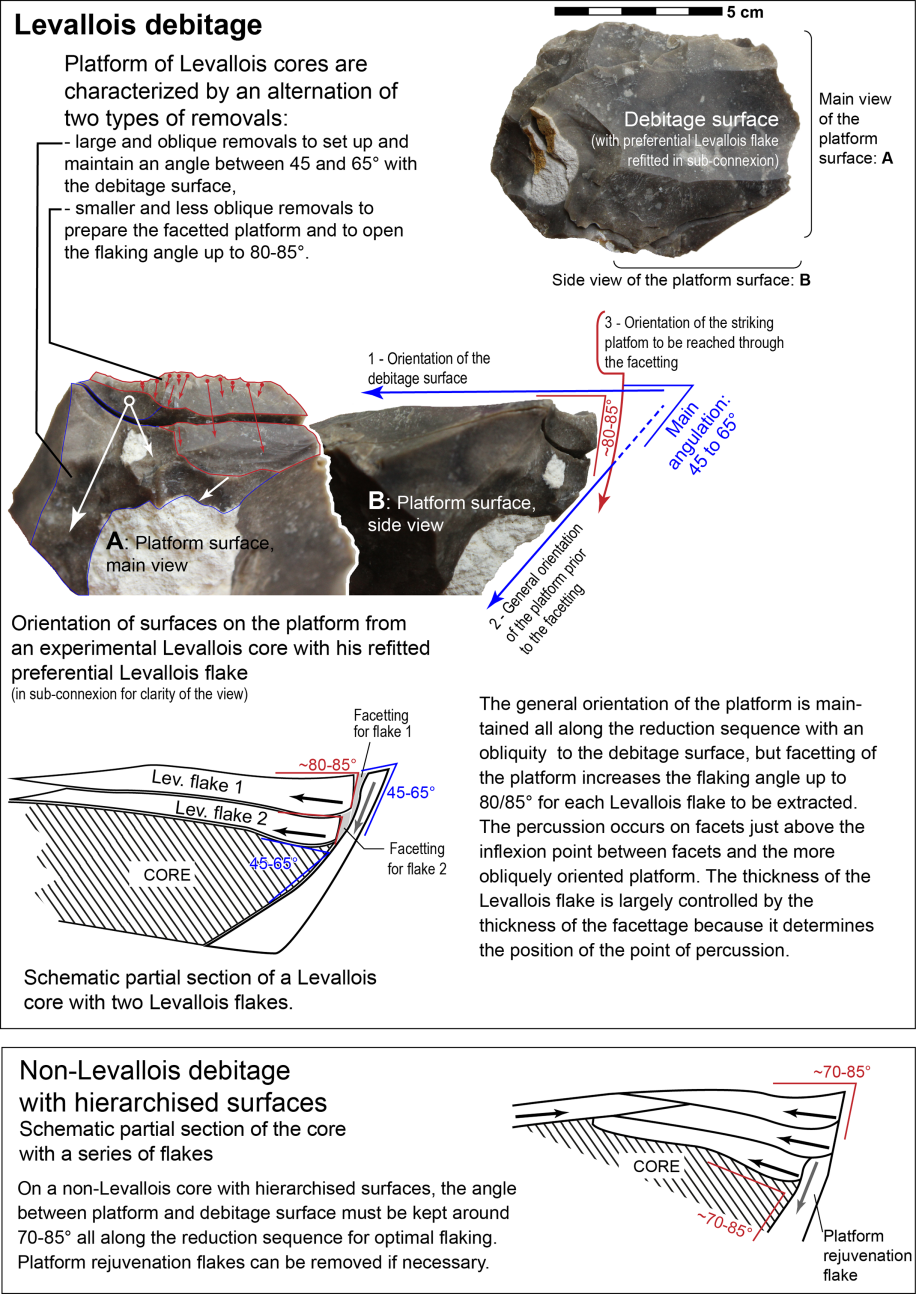
Differences in platform management between Levallois cores and non-Levallois cores with two asymmetric hierarchized surfaces. Levallois platform must allow both the extraction of flakes oblique with respect to the plane of intersection to prepare and maintain convexities and Levallois flakes parallel or sub-parallel to the plane of intersection of the two surfaces. This is a key criterion to distinguish Levallois and non-Levallois cores.

At a first level of reading, the organization of removals on the cores from Guado San Nicola classified as Levallois (7.58% of the cores) by Peretto et al. ([77]: figs 5 and 6) suggest that they could match with the definition of Levallois debitage proposed by Boëda (1994, 1995). They have two asymmetric and hierarchized surfaces and most of these cores have been exploited centripetally or preferentially. With a closer observation of the published materials, the presence of Levallois debitage in the industry from Guado San Nicola appears doubtful. First, there is no step by step presentation of arguments, necessary to support the hypothesis of such an early occurrence of Levallois. For instance, on the schemes of these hypothetical Levallois cores ([77]: fig 6), negatives of flakes on the debitage surface are not distinguished according to their status and orientation, that is, whether they are secant removals shaping the convexities or are subparallel Levallois products and alternation in the flaking chronology between these two types of removals is not documented. It seems that no or limited preparation/maintenance of convexities occurred on these cores. Second, another element defining the Levallois debitage (Boëda, 1994, 1995) but often omitted by researchers is missing here: the management of the striking platform. This surface must allow the extraction of flakes with secant orientation for the preparation or maintenance of convexities and also Levallois flakes parallel/sub-parallel to the core surface (Fig 15). It implies an alternation on the platform between two types of removals. Large oblique flakes (angle of ~45–65° with the debitage surface) will create suitable platforms to extract short flakes with secant orientation to prepare or maintain the convexities. Small and narrow flakes will increase locally up to 80–85° the angle of the platform with the debitage surface to prepare facetted platforms of forthcoming Levallois flakes. This is a key parameter to control the location of the percussion, the transmission of the energy from the hammerstone and the size of the Levallois flake. On Guado San Nicolas cores this alternation is not visible and the angle between the striking platform and the debitage surface seems to be constant and always close to 70–85° ([77]: Fig 5:2, Fig 6:2–4). Thus two of the important criteria of the Levallois debitage are missing on the Guado San Nicola cores. These cores are in fact by-products of a less elaborate centripetal debitage with two hierarchically organised surfaces. For similar reasons, White and Ashton [62] have considered that cores from Purfleet, Botany Pit, dating to either late MIS 9 or early MIS 8, and previously described as ‘proto- or reduced Levallois’ don’t match all the criteria defining Levallois production. We believe that the presence of Levallois debitage in the late MIS 11 Acheulian industry of Guado San Nicola is not conclusively demonstrated.

There is an overall agreement that in Western Europe the Levallois method appears in association with the first manifestations of the Middle Paleolithic. The SdD and MdG occurrence introduces a new degree of diversity within the context of emergence of Levallois production because the industry is significantly different from any one ever described in Western Europe from MIS 9 to the end of MIS 8. The first manifestations of the Levallois technology not only show a variety of methods (recurrent centripetal, recurrent parallel, preferential) but more curiously occur within quite variable industries. In Southwestern France (at Les Bosses about 300 ka or at Raspide 2) the Levallois method (of the recurrent centripetal variety) is less common than other core types (discoidal, simple unprepared cores) and of tools such as bifaces and other large tools (cleavers, choppers) [78,79]. At Orgnac 3, Levallois technology is dominating from level 3 upward with different methods but it is still associated with a few handaxes and pebble tools [80,81]. In England at Baker’s Hole the Levallois debitage from the base of the sequence, dated to the end of MIS 8 and beginning of MIS 7 [82], is represented by the preferential method, occasionally by the recurrent method and is not associated to any biface (except for specimens reworked from older deposits) [83,84]. At Kesselt Op-de-Schans (Belgium), in a level at the transition from MIS 9 to MIS 8, the industry from the ODS1 and ODS2 concentrations is only characterised by recurrent centripetal Levallois debitage incorrectly described as ‘sophisticated discoid technology’ by [85,86]. In Southwestern France, at Petit Bost, in level 2 dated to the end of MIS 9 or early MIS 8, different methods of Levallois debitage, including recurrent parallel uni- or bidirectional, are associated with a few handaxes but also with several cases of Quina debitage [67,87]. These examples suggest that in Western Europe early Levallois occurs in variable technological context. Interestingly the sudden and concomitant emergence of the Levallois method in several European sites in association with a variety of technical repertories -of which SdD and MdG are a clear example- strongly suggest a rapid diffusion over wide geographic spaces of this innovation adopted by groups of different technical traditions thus entering a new technological phase which we call Middle Paleolithic. The SdD and MdG industries are a record of this process.

### The Protopontinian

The SdD/MdG artifact assemblages should no longer be considered as “Protopontinian” even if they share some typological features (a moderately high Quina retouch, Levallois always present but rare, an abundance of sidescrapers) with the so-called Pontinian industries [88] which are dated to the Upper Pleistocene. The structure of the lithic production at SdD/MdG is widely different from the lithic technology of Pontinian assemblages such as Grotta Guattari [30,88,89] whose layers 5, 4 base and 4 are dated to MIS 5, Grotta di Sant’Agostino [90] dated to MIS 3 and Grotta dei Moscerini [30,91] whose sequence spans 123–74 ka (MIS 5) [92].

**Fig 16:**
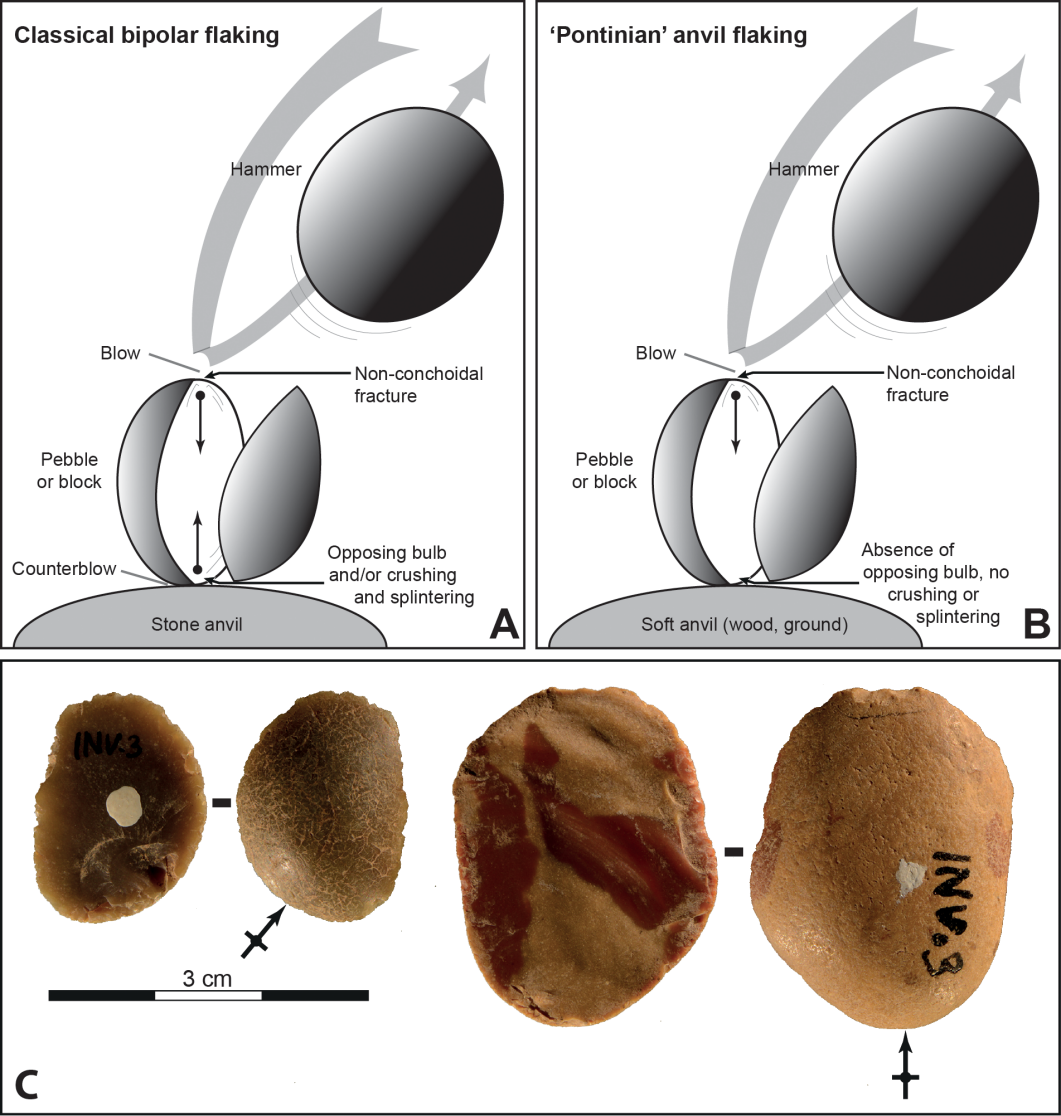
Differences between classical bipolar flaking and ‘Pontinian’ anvil flaking. (A) With the classical bipolar flaking the development of the counterblow results in the formation of an opposing bulb and/or crushing and splintering. (B) With a soft anvil the counterblow doesn’t generate any feature on flakes or cores. (C) Two ‘flat’ flakes from ‘Pontinian’ anvil flaking (Grotta di Sant’Agostino).

Our analyses indicate that all three “Pontinian” assemblages are characterized by the frequent use of a particular variant of the bipolar technique, which is absent at SdD and MdG. The bipolar technique (or hammer and anvil technique) consists in resting a pebble (or a block or a flake) on anvil and striking it with a hammer. This flaking technique can be used to split a pebble in two blanks to be further worked or as a reduction method for producing quantities of flakes and flake fragments from small nodules [25,42–44]. Flakes produced with this technique are characterized by a non-conchoidal fracture, a flat ventral face, a crushed or concave or no bulb of percussion, no measurable platform and an opposing bulb or shattering at the distal end (Fig 16A).

However the flakes of several Pontinian assemblages do not show some features expected on bipolar flakes, such as an opposing bulb, or crushing and splintering at the distal end (Fig 16C). Experiments show that the lack of opposing bulb or other traces at the distal end is due to the use of a soft anvil (e.g. wood) or more simply by resting the pebble on the ground and maintaining it in a vertical position [30,45]. This very specific variant of the bipolar technique (Fig 16B) would result in producing cortical and semicortical flakes with an undamaged distal edge, a significant feature in very small products. We call these flakes “flat” flakes using a terminology first used at the Acheulian site of Terra Amata [93]. However at Terra Amata the limestone pebbles yielding flat flakes were large flat ovals that could be easily held in the hand and experiments [94] show that flat flakes were produced by hand-held vertical percussion on anvil, a mode of production quite different from the knapping of the small flint pebbles of the Latium sites. Flakes without opposing bulb or other counterblow features are also obtained experimentally when a stone anvil is used in bipolar flaking [95] but always in association with bipolar products with blow and counterblow features.

A good proportion of the typical Pontinian sidescrapers were made on flat flakes and sometime on cores with one or several of these flat removals. The ventral face of flat flakes and the flat scars of cores offered a flat and uniform knapping surface. As yet, there are no known antecedents to the Pontinian variant of the bipolar technique in this region. The first use of this particular technique occurs at Grotta dei Moscerini (Table 3).

**Table 3:** Frequency of products and by-products of ‘Pontinian’ on anvil flaking and direct percussion flaking in three Middle Paleolithic assemblages considered as Pontinian.

	Moscerini		Guattari		Sant'Agostino	
	N	%	N	%	N	%
Flat flakes and cores with flat removals	37	*19*.*9*	125	*61*.*3*	192	*37*.*2*
Flakes and cores by direct percussion	149	*80*.*1*	79	*38*.*7*	324	*62*.*8*
Total	186	*100*	204	*100*	516	*100*

Indeterminate flakes and cores, and other kinds of blanks such as chunks, flake fragments, pebbles, and indeterminate are excluded from the counts.

## Conclusions

Present data shows that the SdD/MdG assemblage, securely dated to 295–290 ka, that is close to the time of transition from MIS 9 to MIS 8, is one of the oldest, if not the oldest record of Levallois production in Italy. Despite the small size of the lithic assemblage from SdD and Mdg, most if not all the characters defining the Middle Paleolithic have been identified. Sidescrapers dominate the tool-kit and at least three main chaîne opératoires, including the fully developed Levallois one, have been used to produce blanks of different size, shape and technical properties (involving length and sharpness of retouched or unretouched cutting edges): thin and regular Levallois flakes, slightly elongated flakes and thick ‘core-like’ half pebbles. The Levallois method is clearly associated with a Middle Paleolithic technological context. Direct shaping of unifacial or bifacial tools on small flint pebbles (i.e. chopping tools) is a minor component of the industry and the bipolar percussion is absent. Thus we cannot agree with Picin et al. [65] who believes that the Sedia del Diavolo and Monte delle Gioie industry show a continuity of archaic elements.

The industries from SdD/MdG can no longer be considered as Proto-Pontinian because several technological differences distinguish them from later, Würmian, industries of the so-called Pontinian type and also from the Early Middle Paleolithic from Torre in Pietra level *d*. However a degree of technological continuity can be seen in the selection and use of small flint pebbles and core-like pieces as blanks for retouched small tools.

The SdD and MdG industries show significant differences with broadly contemporaneous industries of the initial Middle Paleolithic with early evidences of Levallois technology such as Orgnac 3, Mesvin IV, Kesselt Op-de-Schans, Petit-Bost, and even with Torre in Pietra level *d* which is more recent and yet geographically very close. Not only the role of the Levallois debitage in the lithic production of SdD/MdG is minor by comparison with those industries; more importantly, the industries of those two sites are characterized by an original debitage method that is actually mid-way between blank production and shaping of a single tool, and intended to produce specific blanks for thick sidescrapers. This technology has never been described before yet is part and parcel of a regional trend to make tools directly from small volumes of raw material -commonly small flint pebbles- as we described it for the Lower Paleolithic industries of Torre in Pietra level *m* and Castel di Guido. This habit indicates a degree of technical continuity between Acheulian industries and Middle Paleolithic industries of the region, including Torre in Pietra level *d*. It is in this context that the Levallois technology was introduced from abroad and successfully integrated in the local technological background on the way to the Middle Paleolithic.

## Supporting information

S1 File Human remains from Sedia del Diavolo and supplementary tables. (PDF)

S2 File History of research at Sedia del Diavolo and Monte delle Gioie. (PDF)

S3 File Fluvial aggradational cycles in the Aniene River Valley. Stratigraphy and chronology of Sedia del Diavolo and Monte delle Gioie.
(PDF)

S4 File Permissions from copyright holders. (PDF)
