# Supplementary material for: Early Levallois and the beginning of the Middle Paleolithic in central Italy
Source: PLoS One. 2017 Oct 20;12(10):e0186082. doi: 10.1371/journal.pone.0186082 (PMC5650164; doi:10.1371/journal.pone.0186082)
Supplement: S1 File — (PDF) [file pone.0186082.s001.pdf]

Supporting Information

Early Levallois and the beginning of the Middle Paleolithic in Central Italy

Sylvain Soriano*, Paola Villa

*To whom correspondence should be addressed. E-mail: sylvain.soriano@cnrs.fr

S1 File. Figures and tables.

This PDF file includes:
- S Figures A-B: Human remains from Sedia del Diavolo
- S Tables A-B: Taphonomy of lithic industry

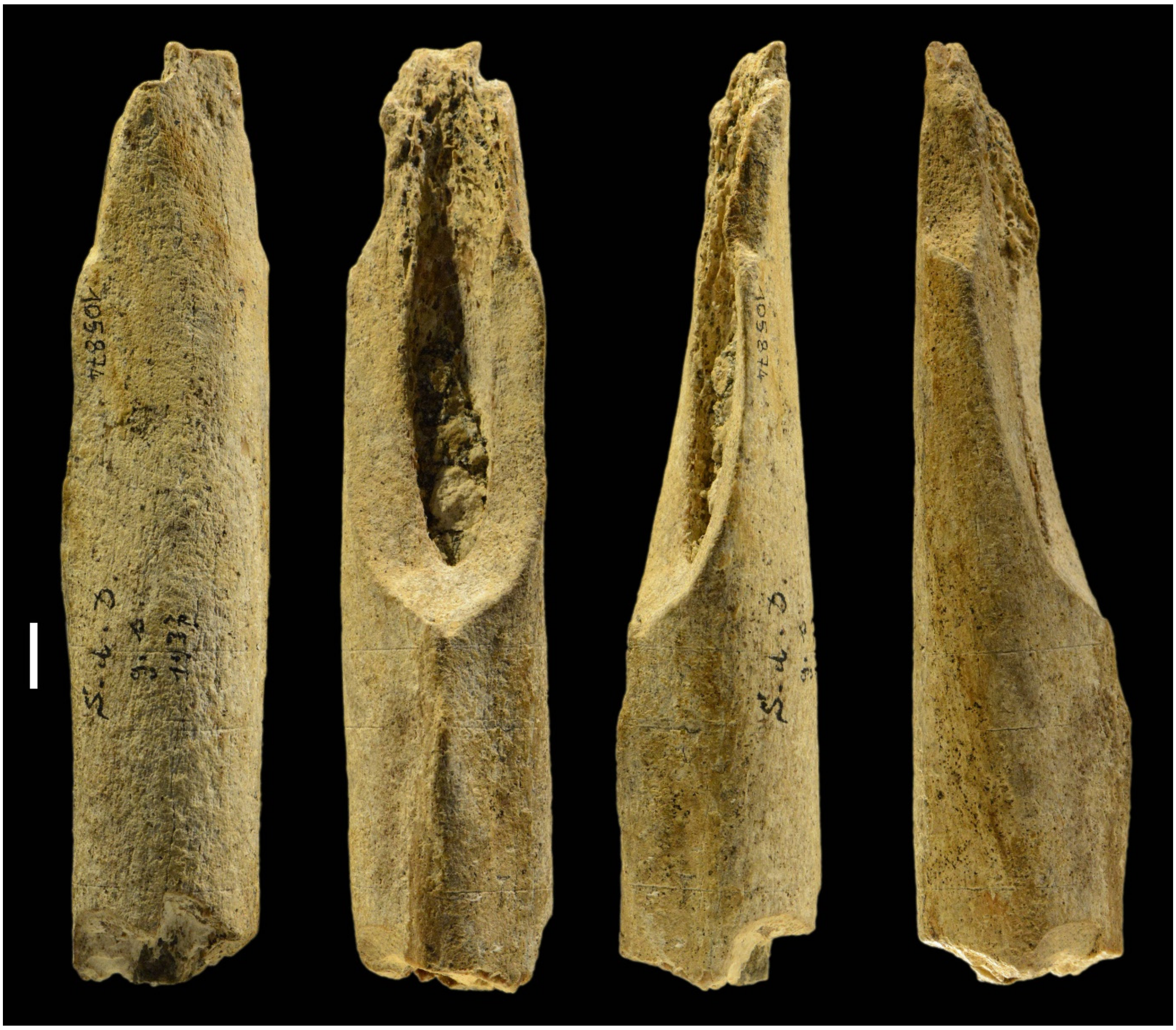

S Figure A. Sedia del Diavolo. Right femoral diaphysis [1]. Scale bar=1cm. Photo Naccari / Pigorini Museum, courtesy of L. Bondioli.

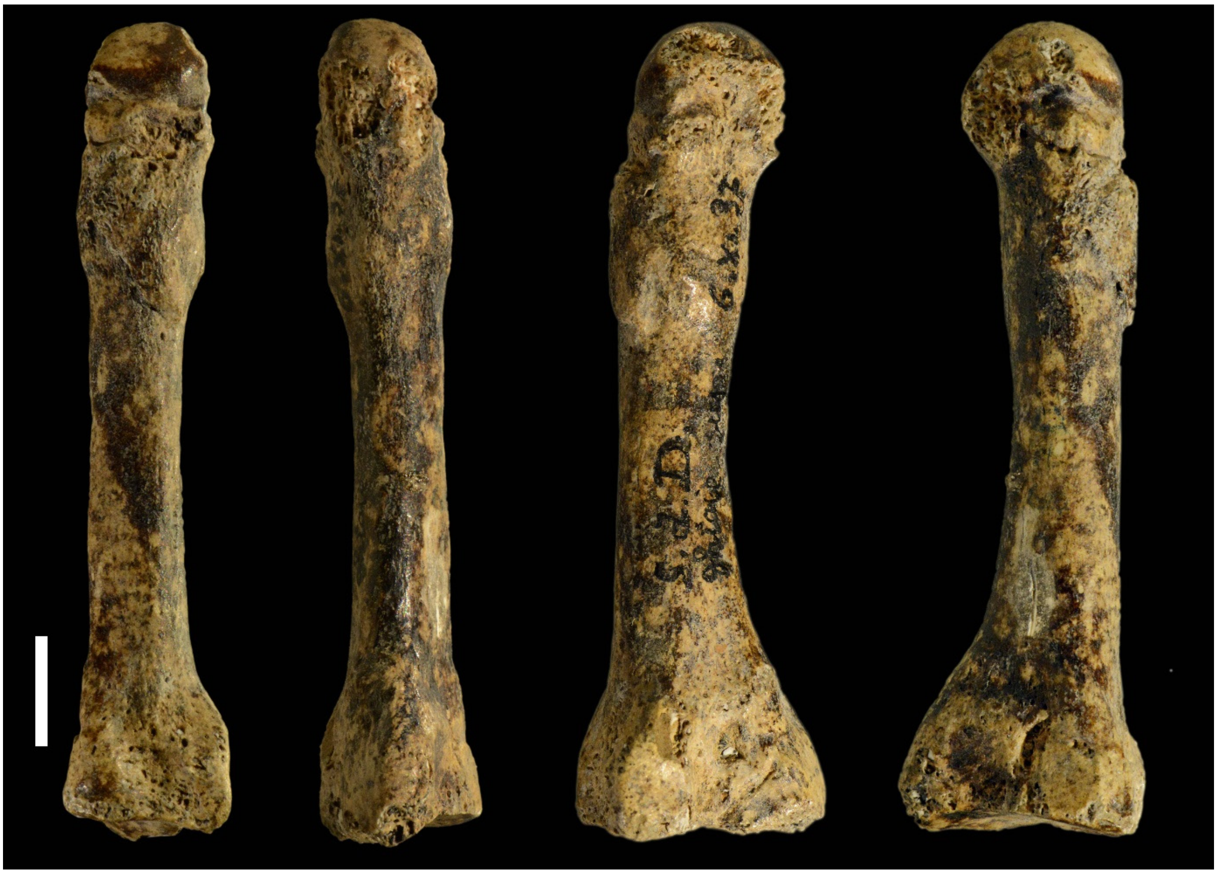

Figure B. Sedia del Diavolo. Metatarsal [1]. Scale bar=1cm. Photo Naccari / Pigorini Museum, courtesy of L. Bondioli.

Table A. State of preservation of artifacts from Sedia del Diavolo and Monte delle Gioie (indetermined cases excluded).

	Fresh	Fluvial polish	Abraded	Very abraded	Total
**N**	90	16	12	2	120
%	*75.0*	*13.3*	*10.0*	*1.7*	*100*

Table B. Mean length, width and thickness of lithic pieces (all types included) from Sedia del Diavolo and Monte delle Gioie.

	Mean Length (mm)	Mean Width (mm)	Mean Thickness (mm)
Sedia del Diavolo	32.9	26.4	10.9
Monte delle Gioie	36.2	25.7	11.3
Total	35.5	25.9	11.2

References

1. Mallegni F. Les restes humains du gisement de Sedia del Diavolo (Rome) au Riss final. L'Anthropologie. 1986;90: 539–553.
