# Supplementary material for: Early Levallois and the beginning of the Middle Paleolithic in central Italy
Source: PLoS One. 2017 Oct 20;12(10):e0186082. doi: 10.1371/journal.pone.0186082 (PMC5650164; doi:10.1371/journal.pone.0186082)
Supplement: S2 File — (PDF) [file pone.0186082.s002.pdf]

Supporting Information

Early Levallois and the beginning of the Middle Paleolithic in Central Italy

Sylvain Soriano*, Paola Villa

*To whom correspondence should be addressed. E-mail: sylvain.soriano@cnrs.fr

S2 File. Text.

This PDF file includes:

Text: History of research at Monte delle Gioie and Sedia del Diavolo.

History of research at Monte delle Gioie and Sedia del Diavolo

The discovery of fossil quaternary faunas at Monte delle Gioie (MdG) and Sedia del Diavolo (SdD) then in the northeast outskirts of Rome, goes back to the end of the 19th century. The hill of MdG (41°56 ' 45 " N; 12°30 ' 44 " E) is on the right bank of the Aniene River, close to its confluence with the Tiber. The first description of the stratigraphy of thick "post-pliocene" deposits at MdG is from Frère Indes who also describes the excavation he have undertaken in a cave developed in the calcareous tufa capping these deposits [1]. This cave which contained plentiful fauna and some lithic remains from an Upper Palaeolithic occupation [2] is referred to as "Indes cave". E. Clerici [3] was the last explorer of this cavity before it was destroyed between 1936 and 1938 by road- and rail work. SdD (41° 55'46"N; 12°31'22"E), on the left bank of the Aniene River 2.5 km upstream, was located on a fluvial terrace portion exploited for gravels and sands that was covered by urban growth of the city of Rome during the 1950's. At SdD the first stratigraphic record was established by R. Meli around 1882.

Taking advantage to ongoing road- and rail work during the 1930's A.C. Blanc recorded stratigraphy at MdG and SdD and in both sites he collected fossil faunas and lithic industry. Similarities between stratigraphy and altitude of gravels deposits were noticed by A.C Blanc et A.G. Segre and these deposits were firmly correlated [5–7]. The last fieldwork was undertaken in April 1956 by A.C. Blanc L. Cardini and P. Cassoli. The stratigraphy published by M. Taschini [4] is a synthesis of previous observations.

Industry and faunas have been recovered at both sites from the bulk of the upper fluvial gravels [4]. Similarities between lithic assemblages from SdD and MdG have been noticed by scholars [4,8,9]. When sorting fauna from SdD, Blanc, Cardini and Cassoli have identified two hominin remains: a metatarsal and a femur fragment described as Neandertal [10–13].

References

1. Indes F. Lettre du frère Indes, sous directeur de l'école Chrétienne à Rome, à M. de Verneuil sur la formation des tufs des environs de cette ville et sur une caverne à ossements. Bulletin de la Société géologique de France. 1869;2ème série, tome 26: 11–28.

2. Segre AG, Segre-Naldini E. 96. Sedia del Diavolo, Lazio. 97. Monte delle Gioie, Lazio. I Primi abitanti d'Europa : 1 500 000-100 000 anni Catalogo della mostra, Museo Nazionale Preistorico Etnografico "Luigi Pigorini", Roma, marzo-luglio 1984. Roma: De Luca Editore; 1984. pp. 200–202.

3. Clerici E. Sopra alcune specie di felini della caverna di Monte delle Gioie presso Roma. Bollettino del Reale Comitato Geologico It. 1888;IX: 149–167.

4. Taschini M. Il "Protopontiniano" rissiano di Sedia del Diavolo e di Monte delle Gioie (Roma). Quaternaria. 1967;9: 301–319.

5. Blanc AC. Notizie sui trovamenti e sul giacimento di Saccopastore e sulla sua posizione nel Pleistocene laziale. Palaeontographia Italica. 1948;XLII (Nuova Serie, XII): 1–24.

6. Segre AG. Sulla stratigrafia dell'antica cava di Saccopastore presso Roma. Rendiconti Accademia Nazionale Lincei (Cl Scienze FMN). 1948;4 (s. 8): 743–751.

7. Segre AG. Geologia quaternaria e Paleolitico nella bassa valle dell'Aniene, Roma in L'Uomo di Saccopastore e il suo ambiente. I Neandertaliani nel Lazio. Celebrazioni per il centenario della morte di C. Darwin. Rivista di Antropologia Roma. 1983;62: 87–98.

8. Palma di Cesnola A. Le Paléolithique inférieur et moyen en Italie. Grenoble: Ed. Jérôme Millon; 1996.

9. Piperno M, Segre A. The transition from Lower to Middle Palaeolithic in Central Italy: an example from Latium. In: Ronen A, editor. The Transition from Lower to Middle Palaeolithic and the origin of modern man. Oxford: B.A.R; 1982. pp. 203–221.

10. Blanc AC. Il più antico reperto fossile umano del Lazio rinvenuto a Roma: un secondo metatarsale destro umano ed industria paleolithica nelle ghiaie superiori della Sedia del Diavolo (Roma). Quaternaria. 1956;3: 342–344.

11. Del Prete A. La diaphyse fémorale dans le genre Homo: variations morphométriques et implications fonctionnelles [Internet]. Thèse de Doctorat, Université de Bordeaux 1. 2000.

12. Mallegni F. Les restes humains du gisement de Sedia del Diavolo (Rome) au Riss final. L'Anthropologie. 1986;90: 539–553.

13. Rossi A. Studio del II metatarsale e di un frammento di femore umani rinvenuti nel sedimento delle ghiaie superiori della Sedia del Diavolo (Roma), pertinente alla glaciazione Nomentana. Quaternaria. 1961;5: 342–344.
