# Supplementary material for: Early Levallois and the beginning of the Middle Paleolithic in central Italy
Source: PLoS One. 2017 Oct 20;12(10):e0186082. doi: 10.1371/journal.pone.0186082 (PMC5650164; doi:10.1371/journal.pone.0186082)
Supplement: S3 File — Stratigraphy and chronology of Sedia del Diavolo and Monte delle Gioie. (PDF) [file pone.0186082.s003.pdf]

Supporting Information

Early Levallois and the beginning of the Middle Paleolithic in Central Italy

Sylvain Soriano*, Paola Villa

*To whom correspondence should be addressed. E-mail: sylvain.soriano@cnrs.fr

S3 File. Text and figure.

This PDF file includes:
    Text and S Figure A: Fluvial aggradational cycle in the Aniene River valley.
    Text: Stratigraphy and chronology from Sedia del Diavolo and Monte delle Gioie.

Fluvial aggradational cycle in the Aniene River Valley

The cyclical structure of quaternary fluvial deposits has been described throughout the world and especially in NW Europe (Somme, Seine, Thames, Escaut, Rhine...). In most of the cases, it is considered to be a climatically controlled process [1,2]. Aggradational cycles (deposition of coarser to finer sediments, downcutting) are controlled by cyclical oscillation of sea-levels induced by global climate changes from glacial to interglacial periods and terraces staircases are records of bedrock uplift. Differences in the timing of downcutting and gravel accumulation have been described depending on several factors. In the lower Thames the downcutting has occurred during cold-to-warm transitions according to [3] whereas in other west-european valleys (ie. Somme [4], Moselle [5]) the downcutting event has occurred at the warm-to-cold transition.
In the Aniene River Valley the aggradational cycle can be described as follows (S Fig A) [6–8]:
(1) At the end of the interglacial, cooling induces sea level lowering. The profile of fluvial drainage is unbalanced and fluvial downcutting begins (2). Previous fluvial deposits are now in a terrace position. Downcutting is enhanced by the transport of coarse materials coming from upstream but no deposition occurs along the course. (3) With cooling that begins after the glacial maximum, the level of precipitation increases. Sea level is rising once again and the base level of the drainage is also rising. Down cutting is now impossible and a rapid accumulation of coarse deposits (boulders, gravels) occur (4) between the glacial maximum and the glacial termination. As the sea level is still rising with warming (5), the gradient of the stream is reducing and only fine sediments (6) can be transported and deposited (sand then silts), sometimes in lacustrine/lagoonal context when the seashore is close. This aggradational sequence is repeated each time the change of sea level under glacio-eustatic control is enough to induce a significant modification of the base level of the drainage.
This model of aggradational sequence applies overall to the Tiber River and its tributaries including the Aniene River but irregular and brutal input of huge amount of volcano-clastic sediments in the fluvial system have induced great variations of thickness, rhythm and nature of deposits. From an archaeological point of view, it implies that most of the glacial time is not recorded. Industries recovered in gravels in rather mint conditions (not too rolled) were deposited in a narrow time span as the gravel accumulation is rapid.

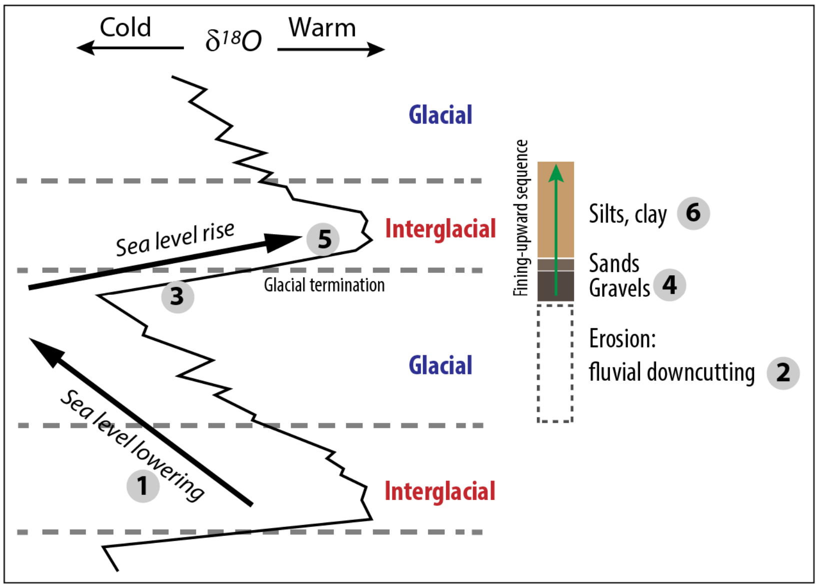

S Figure A. Model of a glacio-eustatically controlled fluvial aggradational sequence in the Aniene River Valley. According to [6–9].

Stratigraphy and chronology of Sedia del Diavolo and Monte delle Gioie

The preservation of two Pleistocene terraces in the Aniene River Valley was first described by A. Segre [10] and A.C. Blanc [11–13]. The latest terrace, referred to as Lower Terrace, is famous for the discovery of two Neanderthal cranium in fluvial sand and gravels at Saccopastore [14] associated with fauna and Mousterian industry. This Lower Terrace was first assigned to the Last Interglacial according to fauna and fossil flora recovered in calcareous tufa [11,15] but is now dated to MIS 7 [6]. An earlier terrace, referred as intermediate [16] or median terrace [17], including pyroclastic deposits from the Latial volcanoes interbedded with gravels, sands and fluvio-lacustrine silts have been observed on both sides of the valley at Monte delle Gioie and Sedia del Diavolo. This median terrace was considered to be rissian [11,18]. The same chronological interpretation was presented in Piperno and Segre [16].

Stratigraphy from Sedia del Diavolo (hereafter SdD), Via Mascagni (Circonvallazione Salaria outcrop) and Monte delle Gioie (MdG) can be described as follows (from base to top) according to data from [12,17–21] (see Fig 2):

- Layer 1: Clay with remains of Conifer only observed at MdG. It might be part of a Paleo-Tiber Unit [9]. Above, a thin layer of clay with terrestrial malacofauna is noticed by [17].
- Layer 2: Fluvial gravels without derived volcanic products. At MdG they outcrop around 2 m thick [19]. At Via Mascagni they were only observed by coring and reportedly contained red scoriae [21].
- Layer 3: Thick (more than 25 m at SdD, ca. 12 m at MdG) deposit of Tufo Lionato, a massive volcanic tuff (ignimbrite) from theAlban Hills volcano dated by $^{40}Ar/^{39}Ar$ to 365 ± 4 ka [22,23]. It is constituted of greyish scoria and yellow-orange pumice

scattered in a cineritic matrix. Three superimposed facies have been distinguished. This layer rests on an erosion surface that cuts underlying gravels.

- Layer 4: Pale yellow silts and sands, fluvial epiclastic deposits with pebbles of reworked Tufo Rosso a Scorie Nere. This layer is 1 m thick at SdD and Via Mascagni.
- Layer 5: Fluvio-lacustrine silts and clays (4 m thick at Via Mascagni, close to 10 m at MdG).
- Layer 6: Fluvial sands (lateral variation of layer 5?). Layers 5 and 6 are locally deeply eroded.
- Layer 7: Fluvial gravels (4 m thick at MdG) resting in unconformity on previous layers and starting a new aggradational sequence. The lower part (1 m) contains pebbles of different derived volcanic materials (ignimbrite with black scoria, Tufo Lionato, pumice). The lithic industry was collected at mid-height of this gravel layer according to [17].
- Layer 8: Lacustrine yellow silty-sands with travertine and tuffite.
- Layer 9: Massive pyroclastic deposit called Tufo Giallo di Sacrofano dated to 285 ± 2 ka [24] originating from the Monti Sabatini Volcanic District. White centimetre-sized pumices are embedded in a grey-brown cineritic matrix.
- Layer 10: Marl with palustrean flora, calcareous encrustations with Typha and Cyperacea.
- Layer 11: On hillside of MdG, a travertine developed and later a cave (Indes cave) formed. The infill of this cave now destroyed contained a rich Upper Pleistocene fauna and an Upper Paleolithic industry [25].

The basal gravels with red scoriae (layer 2) outcropping at SdD and MdG and recognised by coring in the Via Mascagni sequence [21] are difficult to interpret. The red scoriae present in the gravels may be reworked from the Pozzolane Rosse, a pyroclastic flow from the Alban Hills volcanic district, with purple-red scoriae, dated to 456 ± ka [23,26]. Usually, the Tufo Lionato directly underlies the aggradational sequence of the Aurelia Formation [27] and rests on eroded older (MIS 11) deposits. As explained by Karner and Marra [28], in the Fosso Galeria the deposition of the Tufo Lionato was massive (>20 m thick) and fast (faunal remains in anatomical connexion embedded at the base). It occurs during MIS 10 in a fluvial channel already deeply embanked but still downcutting older deposits. We can consider that these gravels are remains from an earlier aggradational phase, separated from the Tufo Lionato by a major unconformity. Fluvial deposits follows, partly reworking the Tufo Lionato (layer 4) then fining-upward deposits (layers 5 and 6) are ending the fluvial aggradational sequence. Layer 7 with lithic industry and fauna, is separated from previous deposits by an erosional surface. It is the first layer of the following aggradational sequence, the so-called 'Via Mascagni succession'. Silty-sands with travertine (layer 8) cap fluvial infilling but they are buried by the Tufo Giallo di Sacrofano, a massive pyroclastic deposit, dated by 40Ar/39Ar to 285 ± 2 ka [23,24].

Thus the Paleolithic artifacts, fauna and hominin remains of Sedia del Diavolo and Monte delle Gioie are time bracketed between two massive volcano-clastic deposits, widely distributed in the area of Rome [23,26]. They are well characterized and well dated by 40Ar/39Ar [22–24]. The age of deposition of layer 7 can be refined taking into account the modelling of pleistocene fluvial aggradation of the Tiber and its tributaries according to Marra et al. [7,8]. Coarse deposits (boulders, gravels) from the base of the sequence are considered

to have been accumulated between the glacial maximum and the glacial termination (See section above). So the lithic industries of Sedia del Diavolo and Monte delle Gioie and hominin remains from Sedia del Diavolo were deposited between the glacial maximum of the MIS 8.6 cold event and the onset of warmer MIS 8.5 episode[1].

References

1. Bridgland D, Westaway R. Climatically controlled river terrace staircases: A worldwide Quaternary phenomenon. Geomorphology. 2008;98: 285–315. doi:10.1016/j.geomorph.2006.12.032

2. Bridgland DR, Westaway R. Quaternary fluvial archives and landscape evolution: a global synthesis. Proceedings of the Geologists' Association. 2014;125: 600–629. doi:10.1016/j.pgeola.2014.10.009

3. Bridgland DR. River terrace systems in north-west Europe : an archive of environmental change, uplift and early human occupation. Quaternary Science Reviews. 2000;19: 1293–1303.

4. Antoine P, Limondin Lozouet N, Chaussé C, Lautridou J-P, Pastre J-F, Auguste P, et al. Pleistocene fluvial terraces from northern France (Seine, Yonne, Somme): synthesis, and new results from interglacial deposits. Quaternary Science Reviews. 2007;26: 2701–2723. doi:10.1016/j.quascirev.2006.01.036

5. Cordier S, Harmand D, Frechen M, Beiner M. Fluvial system response to Middle and Upper Pleistocene climate change in the Meurthe and Moselle valleys (Eastern Paris Basin and Rhenish Massif). Quaternary Science Reviews. 2006;25: 1460–1474. doi:10.1016/j.quascirev.2005.11.007

6. Marra F, Ceruleo P, Jicha B, Pandolfi L, Petronio C, Salari L. A new age within MIS 7 for the Homo neanderthalensis of Saccopastore in the glacio-eustatically forced sedimentary successions of the Aniene River Valley, Rome. Quaternary Science Reviews. 2015;129: 260–274. doi:10.1016/j.quascirev.2015.10.027

7. Marra F, Rohling EJ, Florindo F, Jicha B, Nomade S, Pereira A, et al. Independent 40Ar/39Ar and 14C age constraints on the last five glacial terminations from the aggradational successions of the Tiber River, Rome (Italy). Earth and Planetary Science Letters. 2016;449: 105–117. doi:10.1016/j.epsl.2016.05.037

8. Marra F, Ceruleo P, Pandolfi L, Petronio C, Rolfo MF, Salari L. The Aggradational Successions of the Aniene River Valley in Rome: Age Constraints to Early Neanderthal Presence in Europe. Petraglia MD, editor. PLOS ONE. 2017;12: e0170434. doi:10.1371/journal.pone.0170434

9. Marra F, Florindo F. The subsurface geology of Rome: Sedimentary processes, sea-level changes and astronomical forcing. Earth-Science Reviews. 2014;136: 1–20. doi:10.1016/j.earscirev.2014.05.001

10. Segre AG. Sulla stratigrafia dell'antica cava di Saccopastore presso Roma. Rendiconti Accademia Nazionale Lincei (Cl Scienze FMN). 1948;4 (s. 8): 743–751.

11. Blanc AC. Notizie sui trovamenti e sul giacimento di Saccopastore e sulla sua posizione nel Pleistocene laziale. Palaeontographia Italica. 1948;XLII (Nuova Serie, XII): 1–24.

[1] There are discrepancies in naming these climatic oscillations as pointed by [29]. The latest warm event following MIS 9 temperate optimum can equally be named MIS 8.5 or MIS 9a.

12. Blanc AC. Ricerche sul quaternario laziale. III–Avifauna artica, crioturbazioni e testimonianze di soliflussi nel Pleistocene medio-superiore di Roma e di Torre in Pietra. Il Periodo Glaciale Nomentano nel quadro della serie di glaciazioni riconosciute nel Lazio. Quaternaria. 1955;2: 187–200.

13. Blanc AC. Il più antico reperto fossile umano del Lazio rinvenuto a Roma: un secondo metatarsale destro umano ed industria paleolithica nelle ghiaie superiori della Sedia del Diavolo (Roma). Quaternaria. 1956;3: 342–344.

14. Sergi S. Il cranio del secondo paleontropo di Saccopastore. Palaeontographia Italica. 1946;XLII (Nuova Serie, XII): 25–164.

15. Tongiorgi E. La flora fossile di Saccopastore: suo significato nella storia della vegetazione nella regione laziale. Rivista di Antropologia Roma. 1939;32: 237–242.

16. Piperno M, Segre A. The transition from Lower to Middle Palaeolithic in Central Italy: an example from Latium. In: Ronen A, editor. The Transition from Lower to Middle Palaeolithic and the origin of modern man. Oxford: B.A.R; 1982. pp. 203–221.

17. Segre AG, Segre-Naldini E. 96. Sedia del Diavolo, Lazio. 97. Monte delle Gioie, Lazio. I Primi abitanti d'Europa : 1 500 000-100 000 anni Catalogo della mostra, Museo Nazionale Preistorico Etnografico "Luigi Pigorini", Roma, marzo-luglio 1984. Roma: De Luca Editore; 1984. pp. 200–202.

18. Taschini M. Il "Protopontiniano" rissiano di Sedia del Diavolo e di Monte delle Gioie (Roma). Quaternaria. 1967;9: 301–319.

19. Caloi L, Palombo MR, Petronio C. La fauna quaternaria di Sedia del Diavolo (Roma). Quaternaria. 1980;22: 177–209.

20. Caloi L, Palombo MR, Zarlenga F. Late-Middle Pleistocene Mammal Faunas of Latium (Central Italy): Stratigraphy and Environment. Quaternary International. 1998;47–48: 77–86. doi:10.1016/S1040-6182(97)00073-6

21. Palombo MR, Milli S, Rosa C. Remarks on the biochronology of the late Middle Pleistocene mammalian faunal complexes of the Campagna Romana (Latium, Italy). Geologica Romana. 2004;37: 135–143.

22. Marra F, Karner DB, Freda C, Gaeta M, Renne P. Large mafic eruptions at Alban Hills Volcanic District (Central Italy): Chronostratigraphy, petrography and eruptive behavior. Journal of Volcanology and Geothermal Research. 2009;179: 217–232. doi:10.1016/j.jvolgeores.2008.11.009

23. Luberti GM, Marra F, Florindo F. A review of the stratigraphy of Rome (Italy) according to geochronologically and paleomagnetically constrained aggradational successions, glacio-eustatic forcing and volcano-tectonic processes. Quaternary International. 2017;438: 40–67. doi:10.1016/j.quaint.2017.01.044

24. Karner DB, Marra F, Renne PR. The history of the Monti Sabatini and Alban Hills volcanoes: groundwork for assessing volcanic-tectonic hazards for Rome. Journal of Volcanology and Geothermal Research. 2001;107: 185–215.

25. Indes F. Lettre du frère Indes, sous directeur de l'école Chrétienne à Rome, à M. de Verneuil sur la formation des tufs des environs de cette ville et sur une caverne à ossements. Bulletin de la Société géologique de France. 1869;2ème série, tome 26: 11–28.

26. Marra F, Rosa C. Stratigrafia e assetto geologico dell'area romana. Memorie Descrittive della Carta Geologica d'Italia. 1995;50: 49–118.

27. Conato V, Esu D, Malatesta A, Zarlenga F. New data on the Pleistocene of Rome. Quaternaria. 1980;22: 131–176.

28. Karner DB, Marra F. Correlation of fluviodeltaic aggradational sections with glacial climate history: A revision of the Pleistocene stratigraphy of Rome. Geological Society of America Bulletin. 1998;110: 748–758.

29. Railsback LB, Gibbard PL, Head MJ, Voarintsoa NRG, Toucanne S. An optimized scheme of lettered marine isotope substages for the last 1.0 million years, and the climatostratigraphic nature of isotope stages and substages. Quaternary Science Reviews. 2015;111: 94–106. doi:10.1016/j.quascirev.2015.01.012
