# Supplementary material for: Early Levallois and the beginning of the Middle Paleolithic in central Italy
Source: PLoS One. 2017 Oct 20;12(10):e0186082. doi: 10.1371/journal.pone.0186082 (PMC5650164; doi:10.1371/journal.pone.0186082)
Supplement: S4 File — (PDF) [file pone.0186082.s004.pdf]

Supporting Information

Early Levallois and the beginning of the Middle Paleolithic in Central Italy

Sylvain Soriano*, Paola Villa

*To whom correspondence should be addressed. E-mail: sylvain.soriano@cnrs.fr

S4 File. Permissions from copyright holders.

This PDF file includes:

Permission from Princeton University Press for Figure 1.

Permission from the President of the Italian Institute of Human Palaeontology to use in modified form several drawings from the 1967 M. Taschini paper.

RE: attachment for a submtted request of copyrighted figure

Sujet : RE: attachment for a submtted request of copyrighted figure
De : Permissions <Permissions@press.princeton.edu>
Date : 07/09/2017 20:16
Pour : Paola Villa <paola.villa@colorado.edu>, Permissions <Permissions@press.princeton.edu>
Copie à : "sylvain.soriano@cnrs.fr" <sylvain.soriano@cnrs.fr>

Dear Professor Villa,

Thank you for this additional email.

Your proposed changes to our figure certainly modify the original enough so as to fall within the parameters of Fair Use. We would appreciate if you would include attribution that your new figure is modified from our publication as follows:

Modified from Figure 3.1 in MOUSTERIAN LITHIC TECHNOLOGY: An Ecological Perspective by Steven L. Kuhn. Copyright © 1995 by Princeton University Press.

Thank you for checking with us.

Best,

PUP Permissions Department

Princeton University Press
41 William Street
Princeton, NJ 08540-5237
www.press.princeton.edu
Permissions@press.princeton.edu

Istituto Italiano di Paleontologia Umana
Sede amministrativa: Museo Civico di Zoologia - Via U. Aldrovandi 18, 00197 Roma
Laboratori: Convitto Nazionale Regina Margherita - Piazza Ruggero Bonghi 2, 03012 Anagni (Fr)
Tel. +39 389 0127586 / +39 0775 726091 – E-mail: info@isipu.org ***Web: www.isipu.org***

OGGETTO: Permission to Paola Villa e Sylvain Soriano

Dear Paola and Sylvain,

you have my permission to use in modified form various figures of artifacts published by M. Taschini in Quaternaria 1967 "Il Protopontiniano rissiano di Sedia del Diavolo e Monte delle Gioie (Roma) ". The source must be cited.

Anagni, 20 settembre 2017

Il Presidente
Stefano Grimaldi
